# Environmental Neurotoxin β-*N*-Methylamino-L-alanine (BMAA) as a Widely Occurring Putative Pathogenic Factor in Neurodegenerative Diseases

**DOI:** 10.3390/microorganisms10122418

**Published:** 2022-12-06

**Authors:** Srdjan Lopicic, Zorica Svirčev, Tamara Palanački Malešević, Aleksandar Kopitović, Aleksandra Ivanovska, Jussi Meriluoto

**Affiliations:** 1 Faculty of Medicine, University of Belgrade, Dr Subotića Starijeg 8, 11000 Belgrade, Serbia; 2 Department of Biology and Ecology, Faculty of Sciences, University of Novi Sad, Trg Dositeja Obradovića 2, 21000 Novi Sad, Serbia; 3 Faculty of Science and Engineering, Biochemistry, Åbo Akademi University, Tykistökatu 6A, 20520 Turku, Finland; 4 Faculty of Medicine, University of Novi Sad, Hajduk Veljkova 3, 21000 Novi Sad, Serbia; 5 Innovation Center of the Faculty of Technology and Metallurgy, University of Belgrade, Karnegijeva 4, 11000 Belgrade, Serbia

**Keywords:** cyanobacteria, β-*N*-methylamino-L-alanine, BMAA, neurodegenerative diseases

## Abstract

In the present review we have discussed the occurrence of β-*N*-methylamino-L-alanine (BMAA) and its natural isomers, and the organisms and sample types in which the toxin(s) have been detected. Further, the review discusses general pathogenic mechanisms of neurodegenerative diseases, and how modes of action of BMAA fit in those mechanisms. The biogeography of BMAA occurrence presented here contributes to the planning of epidemiological research based on the geographical distribution of BMAA and human exposure. Analysis of BMAA mechanisms in relation to pathogenic processes of neurodegeneration is used to critically assess the potential significance of the amino acid as well as to identify gaps in our understanding. Taken together, these two approaches provide the basis for the discussion on the potential role of BMAA as a secondary factor in neurodegenerative diseases, the rationale for further research and possible directions the research can take, which are outlined in the conclusions.

## 1. Introduction

Some strains of cyanobacteria are known to produce potent hepato-, cyto-, neuro- and/or dermatotoxins and other bioactive compounds [1]. Blooms of cyanobacteria and cyanobacteria-associated health problems have been documented throughout the world [2,3]. Many cyanobacterial poisonings can be traced back to microcystins, cyanobacterial peptide hepatotoxins, tumor promoters and possible carcinogens, which may pose a serious threat to health through contaminated drinking water [4]. Other cyanobacterial toxins (cyanotoxins) include compounds from the groups of cylindrospermopsins (cytotoxins), anatoxins and saxitoxins (neurotoxins) and nodularins (hepatotoxins, tumor promoters and possible carcinogens). All of these toxins have been implicated in cyanobacterial poisonings of either humans or animals.

One interesting compound in the spectrum of cyanobacterial metabolites is the non-proteinogenic amino acid β-*N*-methylamino-L-alanine, abbreviated BMAA. This compound, according to systematic chemical nomenclature (*S*)-2-amino-3-methylaminopropanoic acid (L-BMAA), has the natural isomers (*S*)-2,4-diaminobutyric acid (L-DAB), *N*-(2-aminoethyl)glycine (AEG) and β-amino-*N*-methylalanine (BAMA) [5]. This review concentrates on BMAA which is the best characterised of the compounds. 

BMAA was first observed in cycad seeds some 55 years ago [6], and its occurrence and properties continue to inspire scientists of various disciplines [7]. BMAA has been suggested to play a causal role in amyotrophic lateral sclerosis/Parkinsonism-dementia complex (ALS/PDC), found at an elevated incidence in the Chamorro people of the Pacific island of Guam [8]. The Chamorro people are thought to become exposed to the neurotoxin through the use of cycad seed flour contaminated by BMAA originating from symbiotic cyanobacteria. As BMAA can be found or even biomagnified in certain food web compartments [8,9,10] it is also possible that a major share of the BMAA exposure on Guam occurs through the traditional consumption of cycad-feeding bats. 

Early research pointed to a wide occurrence of BMAA in free-living and symbiotic cyanobacteria in many parts of the world, at up to mg/g DW levels [11]. Another paper from South Africa confirmed the taxonomic ubiquity of BMAA in freshwater cyanobacteria, but the reported toxin concentrations were lower [12]. BMAA was found in combination with additional cyanotoxins in British waterbodies [13]. In contrast, a study involving 62 cyanobacterial samples of worldwide origin found no BMAA in any of the cyanobacterial samples [14]. BMAA has also been reported in other groups of microalgae (Supplementary Table S1). The extent of BMAA production by cyanobacteria and other organisms may not thus be fully understood, but it seems a widely observed phenomenon. Out of 74 publications dealing with BMAA detection and quantification in samples of cyanobacteria or human tissues, only 12 failed to detect a BMAA signal in environmental samples [15]. Less is known about the transfer and possible biomagnification of BMAA in food webs. Further, it is not fully understood how much of the toxin occurs in free, protein-bound or soluble-bound forms [16] and whether such a bound toxin is bioavailable. 

Some of the BMAA analytical protocols have been shown to suffer from methodological problems or at least from poor reporting [17], and therefore, especially older literature must be treated with caution. Analytical methods relying on liquid chromatography and fluorescence detection of derivatized BMAA seem to be more prone to report either false positives or an overestimation of BMAA concentration in the studied samples. More selective and sufficiently validated analytical protocols involving appropriate sample preparation and relying on liquid chromatography and tandem mass spectrometry (MS/MS) for the detection and quantification of (derivatized) BMAA have overcome most, but not all, of the methodological challenges [15,18]. The AOAC-accepted method for BMAA in a cyanobacterial matrix is based on ultra-performance liquid chromatography of AQC-derivatized BMAA and tandem mass spectrometry [19].

As will be shown in this review, the importance of BMAA in neurodegenerative disease continues to be controversial. Shortcomings in exposure assessment and the slow onset of disease (i.e., a delay between low-level exposure and observed pathological effects) obscure the understanding of the role and significance of BMAA in neurodegenerative diseases. While there is strong circumstantial evidence connecting BMAA exposure and ALS/PDC disease in the Chamorro people, there is much less evidence of the toxico-hygienic relevance of BMAA in other environments and settings. BMAA has been reported in the brain tissues of patients dying from ALS/PDC or Alzheimer’s disease [8] but it was not found in the brains of patients with confirmed Alzheimer’s disease in another study [20]. For instance, it is not known whether BMAA should be seen as a causative or a contributing factor in the neurodegenerative process. A critical review concluded that a causal relationship between BMAA and neurodegenerative disease is not supported by existing data [21] but some of the arguments in the review were later refuted [22]. According to the precautionary principle and knowing the possibly grave outcome of exposure to the toxin, it would be logical to try to avoid all exposure to BMAA until a comprehensive exposure assessment, full toxicological data and a medical consensus about the risks are available.

This review first discusses the biogeography of BMAA occurrence in environmental samples and the organisms in which BMAA has been detected. The review then describes general pathogenic mechanisms of neurodegenerative diseases and how BMAA fits into these. Based on the exposure assessment and a medical understanding of the neurodegenerative processes, some conclusions about the role and significance of BMAA in neurodegeneration will be drawn, and possible avenues for further research proposed.

## 2. Occurrence of BMAA

### 2.1. Data Search Strategy

An extensive literature search was performed on the Scopus, PubMed and Web of Science databases to explore the presence of BMAA in ecosystems worldwide, as well as cases of associated human and animal poisonings, and experimental toxicological publications. Searches were performed using the following key words: (“BMAA”, “DAB” or “AEG”) and (“poisoning”, “intoxication”, “incident”, “death”, “mortality”, “health”, “health effects”, “ALS”, “ALS/PDC”, “Alzheimer’s”, “neurodegenerative diseases”, “adverse effects”, “exposure”, “environment”, “cyanobacteria”, “microalgae”, “phytoplankton”, “zooplankton”, “fish”, “bivalvia”, “crustacea”, “gastropoda”, “mammals” or “plants”). Papers found through these searches contained some additional relevant references.

### 2.2. Occurrence of BMAA in Cyanobacteria and Microalgae

Based on the published papers listed in Supplementary Table S1 (Occurrence of BMAA and its isomers in environmental samples and organisms), BMAA and its isomers (DAB and AEG) were found to be produced by cyanobacteria belonging to 29 genera: *Anabaena* [11,13,14,23,24,25,26], *Anabaenopsis* [26], *Aphanizomenon* [11,13,23,25,27,28], *Calothrix* [11,23,29], *Chlorogloeopsis* [11], *Chroococcidiopsis* [11,26], *Cyanobium* [30,31], *Cylindrospermopsis* [11,14], *Fischerella* [11,26], *Gomphosphaeria* [13], *Leptolyngbya* [27,29,30,31,32,33], *Lyngbya* [11,23,26], *Microcoleus* [30,31], *Microcystis* [11,13,14,23–25, 34], *Myxosarcina* [11,35], *Nodularia* [11,13,14,26,27,30,31], *Nostoc* [8,11,23,26,29,30,31,35,36], *Oscillatoria* [13,26], *Phormidium* [11,26,30,31], *Planktothrix* [11,13,23,25], *Plectonema* [11], *Prochlorococcus* [11], *Pseudanabaena* [13], *Scytonema* [11,23], *Symploca* [11,29], *Synechococcus* [10,11,23,29,30,31], *Synechocystis* [26,30,31], *Trichodesmium* [11] and *Woronichinia* [25]. These cyanobacteria represented: (a) a biomass of cyanobacteria collected from: freshwater (40 reports from different localities), marine (5 reports) and brackish (1 report) environments and (b) cultures of cyanobacterial strains originating from freshwaters (28 strains), marine habitats (14 strains), brackish waters (33 strains), symbiotic plants (10 strains) and terrestrial environments (4 strains). For 32 strains, the origin could not be found in the corresponding publications. There are also some reports about the presence of BMAA and/or its isomers in the biomass of collected cyanobacteria, but the producers were not identified [9,10,37,38,39,40,41,42,43,44].

BMAA and its isomers have been found in 22 supplements made of biomass of the cyanobacteria *Spirulina* [19,36,45] and *Aphanizomenon* [14,46] (Table S1). The compounds have been also detected in cyanobacterial biocrust samples from Qatar [47,48,49,50] and aerosols (air-filters from the lakeshore) [51].

In total, the presence of BMAA and/or its isomers was linked to more than 200 findings related to cyanobacteria from nature (freshwaters, marine and brackish environment, terrestrial habitats and plant symbionts), market samples and specimens from culture collections. Although BMAA and its isomers are found in many ecosystems, the occurrence of these compounds is not ubiquitous. A total of 387 environmental and biological samples (water, fish, aquatic plants) taken from Nebraska (USA) were analysed for BMAA, DABA and anatoxin-a (a compound unrelated to BMAA and DABA). Measurable levels of BMAA, DABA and anatoxin-a were found in 18%, 17% and 12% of the samples, respectively [38]. In a study involving bloom-impacted lakes and reservoirs of Brazil, Canada, France, Mexico and the United Kingdom, 390 samples were taken from 45 lake sampling sites. AEG and DAB isomers were detected in 30% and 43% of the samples, respectively, while BAMA was found in less than 8% of the samples and BMAA was not observed in any sample [37]. No BMAA was found in any of the analyzed biological loess crusts (BLCs, terrestrial samples) taken from various locations in Serbia, China and Iran [52]. These results indicate that while BMAA occurs in many ecosystems, it is not present in every ecosystem. The results also underline the need to look for the isomers of BMAA (DAB, AEG, BAMA) in order to get a more complete picture of the occurrence of the BMAA family of compounds. 

Due to the inconsistency in the methods, the measured concentrations of BMAA have not been reported in this review, but it is only indicated whether BMAA and its isomers have been detected in the samples. It can also be noted that the BMAA and isomer concentrations are not stable in one ecosystem but vary temporally and spatially. They depend on when (daily, monthly and annual variations), where and how the samples have been taken from a waterbody, and how the samples have been analyzed. Further, BMAA can be found in either free or bound forms which necessitate due attention during the analysis and reporting [16]. Comparisons are therefore more meaningful within one publication with a consistent methodology. The physico-chemical environment also seems to have an influence on the BMAA concentration. BMAA in environmental phytoplankton samples ranged from 1 µg/g to 276 μg/g DW, while cultures had higher values ranging from 20 µg/g to 6.4 mg/g DW [53]. One of the most extensive analytical studies on the occurrence of BMAA isomers in bloom-impacted lakes and reservoirs analyzed environmental water samples from five countries [37]. The study did not detect BMAA in any of the samples but observed isomers at the following min-max concentrations: 10–1100 ng/L (DAB), 5–19,000 ng/L (AEG) and 15–56 ng/L (BAMA). The same paper [37] further presented widely varying BMAA isomer concentrations in environmental water samples reported in previously published studies. For instance, the reported BMAA concentrations in environmental waters in the paper [37] and four earlier papers cited therein varied dramatically: not detected, 6.5–7 ng/L (mean values from two years), 10–300 ng/L, 110 ng/L, 1800–25,300 ng/L. The interested reader is advised to consult the papers listed in Table S1 for the reported individual BMAA/isomer concentrations in various sample types and many organisms, but it should be noted that the absolute values reported may not be comparable between the papers.

There are reports that planktonic diatoms (*Bacillariophyta*), dinoflagellates (*Pyrrhophyta*), green algae (*Chlorophyta*), euglenas (*Euglenophyta*), red algae (*Rhodophyta*), *Haptophyta* and *Cryptophyta* also produce BMAA and/or its isomers (Supplementary Table S1). Diatoms are represented by 15 genera: *Achnanthes* [33], *Asterionellopsis* [54], *Aulacoseira* [55], *Chaetoceros* [10,56], *Cyclotella* [55], *Fragilaria* [55], *Halamphora* [54], *Navicula* [33,55], *Odontella* [54], *Phaeodactylum* [10,56], *Proboscia* [33], *Pseudo-nitzschia* [54], *Skeletonema* [10,33], *Tabellaria* [55] and *Thalassiosira* [10,33,56]; dinoflagellates by seven genera: *Alexandrium* [10,54], *Gymnodinium* [57], *Heterocapsa* [54], *Prorocentrum* [54], *Pyrocystis* [54], *Scrippsiella* [54] and *Symbiodinium* [54]; green algae by four genera: *Ostreococcus* [10], *Chlamydomonas* [54], *Chlorella* [54] and *Dunaliella* [54]; *Euglenophyta* and *Rhodophyta* by one genus each: *Eutreptiella* [54] and *Porphyridium* [54], respectively; *Haptophyta* by two genera: *Tisochrysis* [54] and *Emiliana* [54]; and *Cryptophyta* by four genera: *Hemiselmis* [54], *Proteomonas* [54], *Rhinomonas* [54] and *Rhodomonas* [54] isolated from marine and freshwater environments.

### 2.3. Occurrence of BMAA in Animals

BMAA has also been detected in the zooplankton community in the Baltic Sea [9,44]. The presence of BMAA and its isomers in zooplankton organisms, molluscs, crustaceans, fish, birds, mammals and other animals is a consequence of the bioaccumulation of BMAA in food webs [8,41,58].

In the group of molluscs, BMAA was found in bivalvia *Anodonta woodiana* [34], *Antigona lamellaris* [59], *Arca inflate* [59], *Atrina pectinate* [59], *Cerastoderma edule* [57], *Chlamys farreri* [59], *Corbicula fluminea* [34], *Crassosstrea* sp. [59], *Crassosstrea gigas* [29,54,60], *Crassostrea virginica* [61], *Gafrarium tumidum* [59], *Mactra chinensis* [59], *Mercenaria mercenaria* [59], *Moerella iridescens* [59], *Mytilus coruscus* [59], *Mytilus edulis* [9,54,60,62], *Mytilus edulis platensis* [62], *Mytilus galloprovincialis* [10,29,54,59], *Ostrea edulis* [9,27,60], *Periglypta petechialis* [59], *Perna canaliculus* [62], *Perna viridis* [59], *Placopecten magellanicus* [62], *Ruditapes philippinarum* [59], *Scapharca subcrenata* [59], *Sinonovacula constricta* [59], *Solen strictus* [59], *Tegillarca granosa* [59] and an unidentified mussel [63]. BMAA was also found in gastropods *Bellamya aeruginosa* [34], *Neverita didyma* [59], *Neptunea cumingii* [59], *Natica maculosa* [59], *Haliotis discus hannai* [59], *Volutharpa ampullaceal* [59] and *Rapana venosa* [59].

The arthropods (Crustacea) in which BMAA was found are represented by ten species: *Callinectes sapidus* [36,61,64], *Cancer pagurus* [62], *Heterocarpus ensifer* [60], *Eriocheir sinensis* [34], *Macrobrachium nipponense* [34], *Mysis mixta* [44], *Neomysis integer* [44], *Palaemon modestus* [34], *Panulirus* sp. [65] and *Procambarus clarkia* [34].

In the tissues of the fish, there was evidence of accumulation of BMAA after the consumption of BMAA producers, mostly cyanobacteria. A total of 39 species of fish showed the presence of BMAA in their tissues: *Abramis brama* [57], *Anguilla anguilla* [57], *Aristichthys nobilis* [34], *Carassius auratus* [34], *Carcharhinus acronotus* [66,67], *Carcharhinus leucas* [66,67], *Carcharhinus limbatus* [66,67], *Clupea harengus* [9,60], *Coilia ectenes taihuensis* [34], *Coregonus lavaretus* [9,27], *Cyprinus carpio* [34,51], *Erythroculter ilishaeformis* [34], *Esox lucius* [57], *Galeocerdo cuvier* [67], *Ginglymostoma cirratum* [66,67], *Gymnocephalus cernua* [57], *Hemiramphus kurumeus* [34], *Hypophthalmichthys molitrix* [34], *Neosalanx taihuensis* [34], *Negaprion brevirostris* [66,67], *Osmerus eperlanus* [9], *Parabramis pekinensis* [34], *Parasilurus asotus* [34], *Pelteobagrus fulvidraco* [34], *Perca fluviatilis* [57], *Pleuronectes platessa* [60], *Protosalanx hyalocranius* [34], *Pseudorasbora parva* [34], *Rhizoprionodon terraenovae* [67], *Rhodeus sinensis* [34], *Rutilus rutilus* [57], *Salvelinus alpinus* [60], *Sander lucioperca* [57], *Scophthalmus maximus* [9], *Sphyrna mokarran* [66,67], *Sphyrna tiburo* [66,67], *Sphyrna zygaena* [67], *Tinca tinca* [57] and *Triglopsis quadricornis* [9]. Fish samples from Nebraska reservoirs (carp, white crappie, bass, shad, walleye, catfish, wiper and bluegill) showed the presence of BMAA, and in many samples, also the presence of DAB [38]. BMAA, DAB and/or AEG were found in 16 fish-based dietary supplements (shark cartilage powders) from seven manufacturers [68].

The reports about mammals showed that BMAA was detected in flying foxes [41,69], in dolphins [70] and in human brain tissue from some patients who died from ALS [8,41,71]. It was also found in human hair [72].

### 2.4. Occurrence of BMAA in Plants

BMAA and/or its isomers were found in parts of the following symbiotic and other plants: *Azolla filiculoides* [8], *Brassica oleracea* [27], *Cycas micronesica* [8,28,41,73], *Cycas revoluta* [14,23,27], *Cycas debaoensis* [45], *Gunnera kauaiensi* [8], *Lathyrus latifolius* [14] and aquatic plants from Nebraska [38]. BMAA was found in flour prepared from the gametophyte of cycad seeds [41].

### 2.5. Exposure to BMAA

The reports presented in Table S1 suggest that exposure to BMAA can occur through the same routes of exposure that are known for other cyanotoxins: through drinking water (e.g., in a case when a cyanobacterial mass development occurs in a drinking water reservoir), recreational activities (e.g., swimming, canoeing or bathing), the aquatic food web, terrestrial plants and animals and food supplements [53,74,75,76]. Synthesized by cyanobacteria and microalgae, BMAA is transported through some food webs in aquatic ecosystems from zooplankton and benthos invertebrates, planktivorous fish, shellfish, snails and crustaceans to carnivorous fish and mammals. Human contact with BMAA is possible through all these food web compartments in aquatic ecosystems. In terrestrial ecosystems, BMAA can be found in some symbioses of cyanobacteria with higher plants, but it can also be transferred from aquatic ecosystems into terrestrial ecosystems through irrigation. As a consequence of irrigation with BMAA-contaminated water, BMAA can be accumulated in plant tissues and thus reach animals and humans. The potential collective exposure burden through different groups of organisms shown in Table S1 might be one part of the explanation behind significant concentrations of BMAA (up to 350 µg/g) in the brain tissues of some patients who died from ALS/PDC and Alzheimer’s disease [8,41,71].

**Figure 1:**
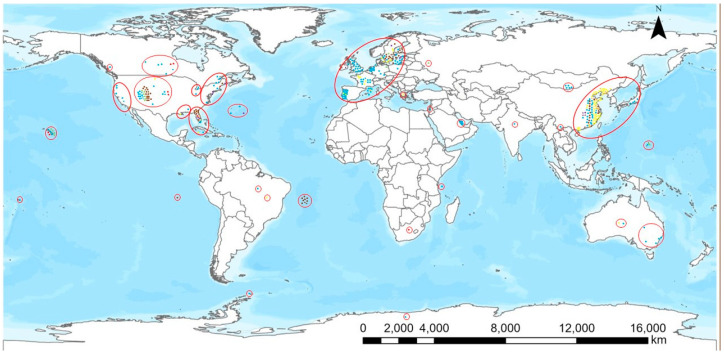
Geographical distribution of the occurrence of BMAA and its isomers. Color coding of the dots: phytoplankton and zooplankton, blue; plants, green; bivalvia, yellow; gastropoda, orange; crustacea, purple; fish, brown; mammals, red.

As presented in Figure 1, BMAA and/or its isomers have been found throughout the world and exposure scenarios either through contaminated drinking water or consumption of contaminated foodstuffs are present in most parts of the world. There are clearly some hotspots of BMAA occurrence: parts of Europe, the United States and China as well as some islands including Guam. The largest number of reports deal with European countries which could be an indication of active research on the topic there. The absence of reports from, e.g., most African, Asian and South American countries probably does not mean the absence of BMAA and its isomers in these parts of the world, but that those territories were not as thoroughly investigated as for instance Western European countries. The situation with BMAA is similar to that of cylindrospermopsin which was first thought to be a tropical toxin. Generally speaking, cylindrospermopsin has been found in most countries where it has been looked for carefully enough and this is the likely scenario with BMAA, too.

According to the map shown, BMAA and also its isomers are frequently recorded in regions where analyses have been performed. Assuming methodological robustness, BMAA and its isomers are thus found in most parts of the world, and therefore there are potential exposure scenarios in many countries. This exposure would be reflected in the incidence of neurodegenerative diseases if BMAA is regarded as a causative factor for such diseases and the exposure levels are high enough. Likewise, the incidence of neurodegenerative diseases would increase upon BMAA exposure even if BMAA is only regarded as a contributing factor in the presence of other risk factors present in the investigated areas. Whether exposure to BMAA in natural conditions really has an effect on the incidence of neurodegenerative diseases has to be proven and confirmed by epidemiological research [2,4].

## 3. BMAA and the Pathogenic Mechanisms of Neurodegenerative Diseases

### 3.1. General Mechanisms of Neurodegeneration

**Figure 2:**
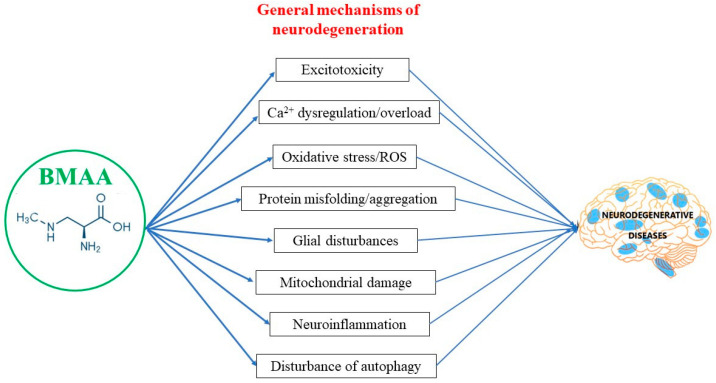
BMAA and general mechanisms of neurodegeneration.

Although neurodegenerative diseases (NDDs) are a heterogeneous group of disorders, each of them affecting distinct anatomical or functional units within the nervous system, many share common pathogenic processes: protein misfolding and aggregation; disorders of proteostasis; mitochondrial damage and oxidative stress; microglial activation and chronic inflammation; and disorders of synaptic function (Figure 2).

The process of protein misfolding and aggregation is one of the hallmarks of NDDs. Protein aggregates are almost invariably present in affected tissues, and often constitute classical pathological criteria for these diseases, even though the proteins involved may differ. In Alzheimer’s disease they are amyloid beta (Aβ), that forms senile plaques, and microtubule-associated protein tau (MAPT, often referred to as just tau) which assembles neurofibrillary tangles; in Parkinson’s disease it is alpha-synuclein which is the primary structural component of Lewy bodies; in amyotrophic lateral sclerosis the main aggregate constituent is TAR DNA-binding protein 43 (TDP-43), etc. Apart from being valuable diagnostic tools, aggregates are also very important in terms of pathogenesis since they often exhibit neurotoxicity and/or trigger other pathogenic processes that lead to neuronal death. For instance, Aβ oligomers may induce mitochondrial dysfunction and oxidative stress, bind to a variety of receptors causing synaptic dysfunction, induce calcium dysregulation, activate intracellular signalling to induce apoptosis, and induce activation of microglia and production of proinflammatory cytokines [77]. They also promote tau phosphorylation, thus fostering tau aggregation into neurofibrillary tangles, which can themselves be neurotoxic. Alpha-synuclein has been shown to have detrimental effects on several intracellular organelles and pathways, including mitochondria, lysosomes, synapses, and microtubules [78]. Finally, aggresomes, aggregates of misfolded TDP-43, may adversely affect neuronal function and viability by causing numerous disturbances such as cellular stress, inhibition of axonal transport, mitochondrial dysfunction, inhibition of endocytosis, and reduced autophagy [79].

Proteostasis is the balance between protein synthesis and degradation in the cell. It is crucial for the maintenance of cellular homeostasis, especially in post-mitotic cells such as neurons. Two main proteostasis mechanisms in charge of altered protein removal are autophagy and the ubiquitin-proteasome system (UPS). Both of these pathways are dysfunctional in most NDDs [80,81]. Since autophagy carries out the degradation of long-lived protein aggregates, and crucially depends on lysosomal function, defects in lysosomal membrane stability, enzymatic content, and activity or acidification of the lysosomal lumen can result in reduced aggregate removal or accumulation of altered degradation pathway intermediates. Autophagy is also involved in organelle dynamics. Therefore, disorders of autophagy affect the neuron’s capacity to maintain proper mitochondrial quality control [82].

In NDDs mitochondria are not affected only by altered autophagy. Protein aggregates can interfere with numerous mitochondrial enzymes, induce disturbances in the respiratory chain complex, and promote free radical generation. This leads to lower mitochondrial ATP production, reduced capacity of mitochondria for calcium handling, excessive production of reactive oxygen species and mitochondrial membrane permeabilization (MMP). All these have deleterious effects on neurons. Reduced energy production interferes with numerous cellular processes in neurons including synaptic assembly and neurotransmission, thus altering synaptic function. Calcium can cause both necrosis and apoptosis, and also damages mitochondrial function, which will be discussed further with excitotoxicity. Excessive mitochondrial ROS generation affects diverse signalling pathways including apoptosis, oxygen sensing, protein kinases, phosphatases, and transcription factors, leading to cell damage and death. Oxidative stress can also stimulate protein misfolding. For instance, in Alzheimer’s disease oxidative stress increases tau phosphorylation. In NDDs, in addition to increased ROS production, the oxidative stress defenses are reduced as well. For example, the altered enzymatic activity of superoxide dismutase has been shown in both PD and ALS. Finally, MMP induces loss of the mitochondrial membrane potential, subsequent respiratory chain uncoupling, reduced ATP synthesis, lysosome disintegration, cell swelling and finally, cell death. As mentioned above, altered lysosome function will affect autophagy which may hinder aggregate removal. In addition, MMP elicits the release of proteins that induce apoptosis and of mitochondrial DNA that plays an important role in neuroinflammation [83,84,85].

The role of microglial activation and neuroinflammation was long considered to be secondary in NDDs, i.e., a reaction to the tissue and cellular damage. Recent research is changing this stance in favour of a more primary pathogenic involvement. The pivotal role in this is attributed to Triggering Receptor Expressed on Myeloid cells 2 (TREM2). TREM2 receptors are selectively expressed in myeloid cells (which are microglia in the brain). They attenuate inflammatory reactions and promote phagocytosis. Mutations of TREM2 have been associated with increased risk for late-onset AD and linked to frontotemporal dementia and ALS. In NDDs, altered TREM2 activation, as well as other causes such as the presence of extracellular mitochondrial DNA secondary to mitochondrial damage and increased mitochondrial membrane permeability, could provoke pathological microglial activation and disturbance of microglial function [86]. Since microglia serve multiple roles in the nervous system, including induction of programmed cell death of neurons, phagocytosis, neuronal plasticity and synaptic pruning, disruption of microglial function could lead to neurodegeneration, reduced removal of aggregates and synaptic malfunction. Aberrant activation of microglia also gives rise to the uncontrolled release of proinflammatory mediators resulting in chronic neuroinflammation [87]. Chronic inflammation in NDDs can be provoked by mitochondrial damage and oxidative stress too. As a consequence of chronic inflammation, there is dysfunction in detecting or responding to the accumulation of protein aggregates, morphological and functional disturbances of the mitochondria, dysregulation of adult neurogenesis, loss of synapses and altered synaptic plasticity [88].

Disturbances of synaptic function on the circuit level result in network instability and dysfunction, while on the single-cell level lead to excitotoxicity. Excitotoxicity is a mechanism of neuronal cell death that is induced by the overactivation of glutamate receptors. The exaggerated activation of glutamate receptors may be a consequence of excessive excitatory neurotransmitter release, reduced excitatory neurotransmitter removal, the presence of exogenous excitatory amino acids, and altered expression or kinetics of glutamate receptors. Whatever the cause, the result is an accumulation of Ca^2+^ in the neuron. This leads to cellular damage and death due to excess stimulation of Ca^2+^-sensitive targets, many of which control key cellular functions. For example, Ca^2+^-dependant activation of calpains is an essential component of necrosis; lipoxygenases are Ca^2+^-activated enzymes that can induce inflammatory response and apoptosis; and a set of DNA-ses involved in apoptosis are Ca^2+^-activated enzymes as well. Furthermore, taking up the excess of cytosolic Ca^2+^ by the mitochondria damages these organelles with consequences that have already been discussed. Finally, many enzymes involved in protein phosphorylation, folding and proteostasis are Ca^2+^ sensitive, which indicates the role that Ca^2+^ overload may have in the formation of protein aggregates [89,90].

All these pathogenic processes in NDDs are inextricably linked. Activation or disturbance of any of them may undermine proper function or promote activation of many others in a feedforward manner so that the effects amplify and the vicious circle of neurodegeneration is formed.

### 3.2. Involvement of BMAA in Pathogenic Mechanisms of Neurodegeneration

Reports from numerous researchers, utilizing various models and routes of administration indicate the potential of BMAA to trigger virtually all of the pathogenic mechanisms associated with primary neurodegeneration (Table 1).

BMAA has been shown to produce a host of synaptic disturbances related to neurotransmitters crucial for neurodegenerative diseases—acetylcholine, which is a key player in Alzheimer’s disease; dopamine, which has a central role in Parkinson’s disease; and glutamate, a neurotransmitter in central motor neurons which are affected by the amyotrophic lateral sclerosis. Rakonczay et al. have reported a significant decrease in acetylcholine esterase activity and glutamate binding in brain homogenates of BMAA-treated rats [100]. Spencer et al. [139] have demonstrated that macaques treated with oral L-dopa, a dopamine replacement therapy, showed selective recovery of marked neurological disturbances induced by BMAA. Interestingly, Lindstrom et al., although reporting dose-dependent lesions of substantia nigra produced by BMAA in rats, have not found any effects on dopamine levels [113]. However, Carion et al. provide evidence that BMAA induces altered expression of genes for the dopamine D4 receptor, indicating that a disturbance in the dopaminergic network may not be transmitter-dependent only but receptor-related as well [94]. Finally, de Munck and colleagues have shown that levels of glutamate are increased and levels of GABA decreased in the motor cortex of rats upon intraperitoneal application of BMAA [106].

Significant as these findings are, from a mechanistic point of view, a more interesting property of BMAA is its potential to trigger excitotoxicity. Indeed, excitotoxicity was the first mechanism of action of BMAA to be hypothesised and investigated. The ability of BMAA to mimic glutamate and act at the glutamate receptors was demonstrated both via reduction or blockade of BMAA-induced subcellular effects and neurotoxicity by glutamate receptor antagonists and in electrophysiological studies.

Attenuation of subcellular and cellular BMAA-induced damage by NMDA ionotropic glutamate receptor (iGluR) antagonists was demonstrated in mice mixed cortical cultures by Ross et al. [117]. Weiss and Choi went on to show that BMAA causes signs of both the acute phase of excitotoxicity (neuronal swelling) and the late phase of excitotoxicity (neurodegeneration), in dissociated mouse cortical cultures. Effects were attenuated by NMDA iGluR antagonists and produced only in presence of bicarbonate ions [137]. Blockade of NMDA iGluRs was also shown to be protective against vacuolisation and swelling of the neuronal somas in chick retina [135].

An early indication that non-NMDA ionotropic glutamate receptors may be involved in the action of BMAA as well came from Weiss et al. They have reported that neuronal cell death in murine mixed cortical cultures induced by BMAA can be prevented by non-NMDA receptor antagonists at lower doses of BMAA (300 µM), and by NMDA receptor antagonists at higher doses of the amino acid (3 mM, [111]). Similarly, and more recently, Liu and co-workers [115] have shown in cultured septal and mesencephalic neurons of mice that NMDA receptor antagonists are protective against neurotoxic effects at higher BMAA doses (500 µM), while at lower BMAA doses (300 µM) the death of neurons is attenuated by non-NMDA receptor antagonists. Activation of non-NMDA iGluRs has also been shown to mitigate BMAA-induced neurological disturbance in mice [116], and neurotoxicity in murine cell cultures [121,131], while NMDA, non-NMDA and metabotropic glutamate receptors (mGluR) were protective against BMAA neurotoxicity in human embryonal and neuroblastoma cell cultures [127].

Electrophysiological studies have provided further evidence of the excitatory nature and excitotoxic potential of BMAA. Weiss and Choi have reported evidence of bicarbonate-dependant rapid membrane depolarisation and increased membrane conductance which were attenuated by NMDA receptor antagonists [137], Wilson et al. have shown that BMAA-elicited depolarisation of field potentials were blocked by NMDA receptor antagonists in rat neocortical slices [130], while Lobner et al. provide evidence that NMDA receptor antagonists attenuate the BMAA induced inward current [121]. Non-NMDA receptors have been implicated in electrophysiological effects too. BMAA-induced membrane depolarisation, increase in intracellular Na^+^ and decrease of intracellular K^+^ concentration of leech Retzius neurons have been partially blocked by non-NMDA receptor antagonists [93]. Additionally, Cucchiaroni et al. conclude that reversible membrane depolarization and inward current of dopaminergic and GABAergic neurons of rat midbrain slices are mediated by the AMPA subtype of non-NMDA iGluRs.

Finally, the previously mentioned finding of de Munck et al. that illustrates an imbalance between glutamate and GABA-favouring excitation [106] indicates that BMAA could induce excitotoxicity not only by direct action on glutamate receptors but also by perturbing the balance between excitatory and inhibitory neurotransmission.

Since Ca^2+^ overload is the principal mechanism by which excitotoxicity induces neuronal injury and death, it is no surprise that in many studies the disturbance of cellular Ca^2+^ homeostasis was investigated together with activation of glutamate receptors. It has been shown that BMAA administration leads to Ca^2+^ influx into the cell and intracellular Ca^2+^ accumulation in neurons [118,121,131,144,147] and glia [133,141]. As mentioned before, in most of these studies Ca^2+^ dysregulation has been linked to the activation of glutamate receptors, but other mechanisms have also been proposed, such as the activation of system Xc^−^ [115] and the sodium/calcium exchanger [148].

BMAA has been shown to induce mitochondrial vacuolisation [103,138], fragmentation [92], and reduced mitochondrial viability [127]. BMAA can also produce endoplasmic reticulum disassembly [92,138]. This is important because the endoplasmic reticulum and mitochondria form mitochondria-associated ER membranes (MAMs), which are involved in calcium homeostasis, mitochondrial dynamics, autophagy, inflammation, and apoptosis. The levels of glutamate dehydrogenase 1 [97], an enzyme implicated in glutamate metabolism and energy homeostasis, as well as glycogen synthase kinase-3 beta (GSK-3β) [138], an enzyme with a role in glucose homeostasis and energy metabolism, inflammation, mitochondrial dysfunction, and apoptotic pathways, are altered as a result of BMAA application too.

As a result of organelle and enzymatic disturbances, BMAA affects numerous cell critical processes. Energy metabolism in neurons is reduced with diminished oxygen consumption, reduced glycolysis and ATP production [145]. Increased production of reactive oxygen species (ROS), and oxidative stress are induced by BMAA in murine neurons from the spinal cord [131], cortex [120,121], neuroblastoma [132] and neuronal stem cell cultures [98], in rat midbrain slices [147] and human cell lines [145]. As indirect evidence of oxidative stress induction and ROS-mediated cellular damage, ROS scavengers have been shown to be protective in BMAA-induced neuronal cell death [115,148]. It has also been demonstrated that BMAA induces ROS generation in the rat [141,143] and human [133] glial cells.

Another process linked to mitochondrial damage is the induction of apoptosis. BMAA induces apoptosis and neuronal loss in the cingulate cortex and the hippocampus of mice [99,140] and rat motoneurons [103] and alters caspases 3 and 9 levels and activity in the rat motoneurons [125,138], cortex, hippocampus, substantia nigra and spinal cord [130]. BMAA can also influence the apoptosis regulator Bax and trigger the unfolded protein response (UPR) mediated apoptosis [110].

As discussed above, a crucial mechanism for the sustainment of healthy mitochondria and the maintenance of mitochondrial dynamics is autophagy. BMAA alters autophagy in rat cerebellar Purkinje cells [92] and affects factors with prominent roles in autophagy such as TRPML1 (transient receptor potential cation channel, mucolipin subfamily, member 1), lysosomal function and sequestosome-1/ubiquitin-binding protein p62 (SQSTM1/p62) in rat motoneurons [125].

Autophagy is significant not only for mitochondrial dynamics, but also for aggregate removal, and therefore disturbances of autophagy favour the accumulation of misfolded protein aggregates. BMAA contributes to this process through aggregate formation as well. It induces enzymes involved in protein misfolding and aggregation [110,134,138,150] and can lead to the accumulation of all relevant protein aggregates—amyloid-beta [114,126,145], tau protein [97,103,114,126,130,150], alpha-synuclein [126] and TDP-43 [92,97,103,126,134,138,143].

Neuroinflammation and microglial activation are common to many neurodegenerative diseases. BMAA stimulates the NLR family pyrin domain containing 3 (NLRP3) protein, a component of inflammasomes that trigger an inflammatory response, increases the activity of caspase-1, an enzyme that initiates inflammation and activates interleukin 1β, an important mediator of inflammation that can promote the spread of inflammation and is also elevated as a result of BMAA application [145]. BMAA causes overexpression of other pro-inflammatory factors as well, such as cyclooxygenase-2 (COX-2), nuclear factor kappa B (NF-κB) and tumor necrosis factor alpha (TNF-α) [103] and can lead to activation of microglia [126].

Finally, BMAA has been shown to alter gene expression and induce genotoxicity in nerve cells [94,97,98,105,107,112,140,144], thus changing the expression of proteins related to mitochondrial dysfunction, apoptosis, ROS handling and proteostasis and creating a milieu that facilitates neurodegeneration.

## 4. Discussion and Conclusions

In the present review we have discussed the biogeography of BMAA occurrence in environmental samples representing different organisms worldwide, and how BMAA fits into general pathogenic mechanisms of neurodegenerative diseases.

BMAA is a widely occurring compound both taxonomically and geographically (Supplementary Table S1). It is present in cyanobacteria from nature (freshwater, marine and brackish environments, terrestrial habitats and plant symbionts), market samples and specimens from culture collections. It has been shown to bioaccumulate or even biomagnify in certain organisms consumed as food. Exposure scenarios are thus present in many parts of the world. As cyanobacteria are generally favoured by the continued discharge of nutrients into waterbodies, rising CO_2_ levels and warmer water temperature [151] it is likely that the frequency and magnitude of cyanobacterial problems will increase in the future.

An analysis of the biomedical literature on BMAA shows that although this review is based on references that are only examples of the indicated mechanisms of action of BMAA (Table 1), and not a comprehensive presentation of all references that show the action of BMAA (according to our records more than 200 papers), it can be easily seen that pure synthetic BMAA was used in most experiments (more than 92% of selected papers). In addition to pure synthetic BMAA, purified BMAA from cycad material, extracts of cyanobacteria, flour of cycads and extracts of cycads were used in BMAA research. Although the toxicity of a substance can be best studied when analysing it in a pure form, there are very rare occasions when people are exposed to pure cyanobacterial toxins, including BMAA. In natural conditions, BMAA is found in a combination with other (secondary) metabolites of cyanobacteria or plants, the potential synergistic action of which is neglected when working with the pure toxin. In the natural sources of BMAA, other compounds that either increase or decrease the toxicity or influence the onset of disease can be found. The importance of these compounds is completely ignored when pure BMAA is utilised in the experiments [152]. Due to such potential interaction of cyanobacteria and cycad biomass extracts, one concentration of BMAA in the extract can be either more or less toxic than the same concentration of pure BMAA. 

In the surveyed studies, the natural route of human exposure was also partially neglected. It is known that the sources of BMAA are the biomass of cyanobacteria (highest during mass occurrence), the biomass of plants (mainly cycads), and animals (to which BMAA has been transferred through food chains). In natural conditions, humans can be exposed to BMAA present in drinking and recreational waters and through food such as aquatic and terrestrial animals, edible plants, and cyanobacteria-based food supplements [8,9,53]. Intended ingestion and accidental ingestion are more relevant for exposure than dermal contact or inhalation. For these reasons, it would be reasonable that the most common route of BMAA exposure would be oral, which was not applied in the majority of analysed papers (Table 1). The oral route of exposure was followed in only 20% of experiments which is equal to the percentage of the subcutaneous injection route. Other exposure routes were also applied in the surveyed investigations: intracerebroventricular (14%), immersion (11%), intraperitoneal (11%), intravenous (9%), inhalation (6%), intracranial (3%), intranasal (3%) and intrathecal (3%). 

The majority of the tested organisms (Table 1) were mammals in the in vivo studies (37%), which is the expected scenario when looking for a more realistic picture of the BMAA role in neurodegenerative diseases. In addition to experiments with mammals, in vivo experiments were done with fish (5%) and arthropods (4%). The remaining 54% of the papers were in vitro studies where cell lines were dominant (34%), then in vitro experiments with mammals (7%), annelids (1%), birds (1%) and enzyme kits (1%). 

The usually chronic nature of BMAA exposure and the delayed mechanisms of BMAA action were typically underestimated in the experiments. Most experiments were done under acute exposure (84% of the surveyed papers) while the emphasis should be on oral, chronic BMAA exposure to environmentally observed concentrations. 

However, even though there are some shortcomings, the research spanning more than thirty years described in this review illustrates that the neurotoxicity of BMAA has been established in a multitude of animal and cellular models, through various routes of administration and involving multiple mechanisms of neuronal damage and death. Yet, the role of this amino acid in the pathogenesis of neurodegenerative diseases remains a matter of controversy and dispute. This dissent can be attributed in large part to the fact that relatively high concentrations of BMAA were required to produce the effects. This indeed is a problem if BMAA is viewed as a sole, or even principal causative agent of neurodegeneration, a stance that has sometimes been taken or proposed. However, this approach may be problematic for two reasons.

Firstly, it is considered that the aetiology and pathogenesis of primary neurodegeneration are multifactorial. The causes are believed to involve genetic, environmental and factors related to aging. These causes trigger multiple mechanisms that are interrelated, and that may affect and amplify each other, forming complex and overlapping vicious circles which finally result in cell dysfunction and death [153]. As an illustration, therapeutic approaches targeting a single one of the pathogenic pathways have mainly shown limited success [154].

The papers analyzed in this review provide evidence that BMAA can initiate and/or facilitate most of the mechanisms related to neurodegeneration. In this regard, even without the potency to cause the disease, by altering the intensity and kinetics of intertwined processes and pathways central to neurodegeneration, BMAA may act as a secondary or contributing factor. In favour of this, Lobner et al. have shown that BMAA at concentrations of 10–100 µmol potentiates neurotoxicity induced by NMDA, Fe, amyloid-β and MPP+ [121]. In other words, in genetically or otherwise susceptible individuals that would develop the disease regardless of exposure, exposure to BMAA would lead to the earlier onset and faster progression of the illness.

The second point is that, as discussed above, BMAA has almost exclusively been used in research alone and as a synthetic compound, even though in nature it often appears together with other environmental factors including, but not limited to, BMAA isomers and metals. Compounds isomeric to BMAA have been shown to be neurotoxic themselves [155], while some metals, such as iron, mercury, lead and aluminium, are also known to contribute to neurodegeneration [156]. It is noteworthy that the presence of metals related to neurotoxicity has been reported in soil and water in the regions of the Western Pacific where a cluster of a high incidence of neurodegenerative diseases, the so-called amyotrophic lateral sclerosis/Parkinsonism dementia complex (ALS/PDC), has first spurred the interest for BMAA as a factor in neurotoxicity [157]. Additionally, the use of the seeds of the Cycas palm is epidemiologically linked to the occurrence of ALS/PDC in affected populations, and BMAA isomers were detected in the seeds of at least one species [14]. Therefore, BMAA could act in concert with other neurotoxic compounds and environmental factors in an additive or synergistic ways.

Although some of these issues have been addressed (e.g., Nunn et al. [158] and Takser et al. [132]), more research is needed to further elucidate the matter. We propose that this further research could be directed at the interactions of BMAA with other environmental factors, as well as the possibility that BMAA acts as a contributing factor that speeds up rather than initiates the pathogenic processes of neurodegeneration, thus not causing the neurodegenerative diseases but leading to their earlier onset and faster progression. In conclusion, the biogeographic and biomedical data in the literature strongly suggest global distribution and risk of human exposure to BMAA, as well as neurotoxicity of this amino acid. Even though this does not mean that BMAA is in any way linked to neurodegenerative diseases, it does justify further research. Risk of human exposure prompts the need for the matter to be resolved, while BMAA remains relevant even as a secondary factor since delayed onset and slower progression of the neurodegenerative diseases would be a significant benefit not only to the patients and their families but also to health and social services. In order to establish whether the presence and neurotoxic properties of BMAA pose a risk to human health, continuing research efforts into the epidemiology and potential role of BMAA in the pathogenesis of neurodegeneration are needed.

## Figures and Tables

**Table 1:** Summary of studies on the effects/mechanisms of BMAA neurotoxicity.

BMAA Source (Cells, Extracts, Purified)	Species/Cell Culture	Route	Dose/Concentrations	Duration	Effects	Reference
Synthetic	Zebrafish (*Danio rerio*) *	i.p.	16.3 µg	0–15 days	Short-term learning and memory deficit	[91]
Synthetic	Rat, Wistar (male) *^/^***	i.p.	300 mg/kg	5 days	Swelling, vacuolisation, fragmentation of endoplasmic reticulum, Golgi desorganisation, unanchored ribosomes, mitochondrial fragmentation and increased autophagy in cerebellar Purkinje cells; cytosolic aggregates of TDP-43.	[92]
Synthetic	Leech **		100 µM, 500 µM, 1 and 3 mM EC_50_: 4 mM ^†^	3 min	iGluR mediated concentration dependent membrane depolarisation, increase in intracellular Na^+^ and decrease of intracellular K^+^ concentration of Retzius neurons.	[93]
Synthetic	Mangrove rivulus larvae *^/^***	Immersion	20 μg/L, 15 mg/L	14 days (0–14 dph)	Altered expression of genes for Dopamine D4 receptor (DRD4), Monoamine oxidase A (MAOA) and Calmodulin (CaM).	[94]
Synthetic	Mice C57BL/6J *^/^**	Intracranial	100 mM	24 h	Death of hippocampal pyramidal neurons	[95]
Synthetic	NSC-34 Cells **		50 µM, 100 µM, 500 µM, and 1000 µM	18 h	Death of neurons	[95]
Synthetic	Mice; Swiss albino brain slices **		1 mM, 5 mM	1 h	Dose-dependent release of LDH and potassium from the slice.	[96]
Synthetic	Zebrafish (*D. rerio*) *^/^***	Immersion	100, 500, or 1000 µM (equivalent to 2.36–23.6 mg/g dry weight of the fish)	6 hpf–6 dpf	Behavioral changes, upregulated expression of CDC-42, glutamate dehydrogenase1 and TDP-43.	[97]
Synthetic	Mice; CD-1IGS female *	Intranasal	50 mg/kg bw	Only dams treated; gestational day 6 to postnatal day 21	No gross toxicity or behavioral changes in dams; sex-specific postnatal development and behavioral alterations present and persistent in offspring.	[98]
Synthetic	Primary neuronal stem cell culture from C57Bl/6J mice **		1, 3, or 10 mM	6 h to 7 days	Increased ROS generation, genotoxicity; no alterations in cell viability.	[98]
Synthetic	Rat, Wistar *^/^***	s.c.	50, 200 or 600 mg/kg	Injected once/day at PNDs 9 and 10; animals than followed for 24 h to 28 weeks.	Induction of apoptosis and neuronal loss in the cingulate cortex and in the hippocampus impairments in spatial learning and memory (open field test, object recognition test, water maze test, elevated plus maze test and radial arm test).	[99]
Synthetic	Rat, Sprague Dawley male *	i.c.v.	500 µg/day	16–60 days	Splay, increased breathing amplitude, pronounced jerking movements, rigidity, hyperactivity, wet-dog shaking, facial tremor, chewing and forepaw grooming	[100]
Synthetic	Rat, Sprague Dawley male brain homogenate **		0.01–10 mM	45–60min	Significant decrease in AChE activity, ^3^H-quinuclinidyl benzilate and ^3^H-Glu binding; inhibition of ^3^H-AMPA binding.	[100]
Synthetic	Zebrafish (*D. rerio*) embryos *	Immersion	0, 5, 50, 500, 5000 or 50,000 µg/L	Exposed for 4 days; observed for 3–9 days.	Abnormal spinal axis formation, clonus-like convulsions.	[101]
Synthetic	Mice, C57/BL male *^/^***	i.v.	3.8 mg/kg bw	0–24 h	High uptake of BMAA to the olfactory tubercle, the cerebral cortex, thalamus, inferior colliculus, brainstem and cerebellum; ~80% of the total ^14^C-L-BMAA in the brain protein-bound.	[102]
Synthetic	Rats, SD male *^/^***	i.v.	300 mg/kg bw	Injected for 3 days followed for 2–8 weeks	Loss of muscle force both in forepaws and hind paws, memory deterioration, pathological changes in nerve conduction, astrogliosis, demyelination, significant loss of axons and dendrites, motor neuronal necrosis and apoptosis, mitochondrial vacuolisation and degeneration, overexpression of pro-inflammatory factors (COX-2, NF-κB and TNF-α), downregulation of GLT-1, cytosolic aggregates of TDP-43 and accumulation of µtubule-associated protein tau	[103]
Synthetic	Rats, Wistar *	s.c.	200 mg/kg/day or 600 mg/kg/day	Injected for two days at pnd 9 and 10; followed for 22 weeks.	Long-term effects on performance of behavioral tasks, especially impairments in spatial learning.	[104]
Synthetic	Rats, Wistar olfactory ensheathing glial cells **		250 µM and 500 µM	48 h	Alterations in expression of genes and synthesis of proteins involved in mitochondrial dysfunction, apoptosis, ROS handling and proteostasis.	[105]
Synthetic	Rats, Wistar male *^/^***	i.p.	300 mg/kg	14 moths	Loss of muscle volume in hind limbs, motor cortex thinning, ventricular enlargement, MRI signs of neurodegeneration in the nucleus of the solitary tract and the motor trigeminal nucleus, increased levels of glutamate and decreased levels of GABA in the motor cortex, loss of depolarization control in the glutamatergic neurons, rise of taurine levels.	[106]
Synthetic	SH-SY5Y cells **		20, 40, 60, 80 mM	60 min	Nitrosated BMAA leads to formation of DNA single strand breaks and cellular toxicity.	[107]
Synthetic	Rats, Sprague–Dawley *^/^***	s.c.	100 mg/kg and 500 mg/kg bw EC_50_: 1mM	Injections on pnd 2 and 5; followed for 101 days.	No behavioral alterations; mild neurochemical changes not indicative of neurodegeneration.	[108]
Synthetic	Mice; Swiss cotical and strital cell cultures **		1 to 1500 μM	48 h	BMAA uptake by cortical cells, cortical neuron cell death at BMAA concentrations > 500 µM, dose-dependent axonal fragmentation, transcellular spread of BMAA to striatal neurons and astrocytes not primarily exposed to the amino acid.	[109]
Synthetic	SH-SY5Y, HT22, Neuro-2a **		1, 2 and 3 mM	1–48 h	Neuronal injury and death in a dose- and time-dependent manner; significant increase of Bax, significant increase in ER chaperons; activation of the ERK, JNK and p38 MAPK pathways; URP signaling mediated apoptosis.	[110]
Synthetic	Mice, mixed cortical cell culture **		10, 100, 300 µM, 1 and 3 mM EC_50_: 1mM	1 h, 1 day and 3 days	Dose- and time- dependent cell death at concentrations >300 µM, cell loss selective to neurons (glia spared), protective effects of non-NMDA antagonists at lower doses of BMAA (300 µM), and of NMDA antagonists at higher doses of BMAA (3 mM).	[111]
Synthetic	Rats, Sprague–Dawley male *^/^***	i.c.v	400 µg	1–4 h	major increase in heat-shock protein 70, c-fos and brain derived neurotrophic factor (BDNF) mRNAs in hippocampus, nerve growth factor mRNA profoundly increased in the dentate gyrus	[112]
Synthetic	Rats, Sprague–Dawley male *^/^***	i.c.v	10 µg, 400 µg	1 week	Dose-dependent lesions of substantia nigra surrounded by profound gliosis, complete loss of TH-immunoreactive neurons in the lesion, no effect on levels of DA, decreased levels of NA.	[113]
Synthetic	Vervet *^/^***	Oral (feeding)	21 and 210 mg/kg/day	140 days	Dose-dependent NFT formation in the perirhinal and entorhinal cortices, amygdala (paralaminar nucleus), motor cortex, frontal cortex, temporopolar cortex and occipital cortex, β-amyloid deposits in the frontal, temporal and motor cortices.	[114]
Synthetic	Mice cultured septal and mesencephalic neurons **		3, 30, 300 mM, and 1 mM	24 h	Neuronal cell death in both septal and mesencephalic cultures, in septal cultures cholinergic neurons significantly more sensitive than general neuronal population, at higher BMAA doses (500 µM) NMDA receptor antagonists and ROS scavengers protective, while at lower BMAA doses (300 µM) death of neurons attenuated by non-NMDA receptor blocker.	[115]
Synthetic	Mice, Swiss Webster male *	i.c.v.	0.5–1.5 µM	2 h	Ataxia, ptosis, rolling, unsteady gait, forelimb clonus, hyperlocomotion, myoclonic jerks, jumping, clonic muscle spasms and hypolocomotion in treated mice, CD50 values for BMAA increased greatly by non-NMDA receptor antagonist.	[116]
Synthetic	Mice, Swiss albino mixed cortical cultures from motor cortex **		0.16–3.2 mM	15 min	Concentration-dependent appearance of edematous vacuoles located postsynaptically, as well as dark and shrunken cells in deep cortical layers. Neurotoxicity attenuated by NMDA receptor antagonist.	[117]
Synthetic	Dissociated brain cells from Sprague /Dawley rats **		5 mM	1 min	Nanomolar increase of intracellular calcium concentration in the presence of bicarbonate.	[118]
Synthetic	*Artemia salina* *Nassula sorex* (*variabilis*) *Danio rerio* *	Immersion	0.05 to 5000 μg/L	24 h–5 days	Increased mortality in all three species, loss of group swarm response, reduced motility, and a significant loss of a positive phototactic locomotory response in adult *A. salina*	[119]
Synthetic	Mice, Swiss Webster mixed cortical cell culture **		3 mM	1 and 3 h	Attenuation of cystine uptake by inhibition of the cystine/glutamate antiporter (system Xc−), decrease of glutathione levels, significant increase in oxidative stress.	[120]
Synthetic	Mice, Swiss Webster mixed cortical cell culture **		10–10,000 µM	1 to 24 h	At concentrations >1 mM, BMAA alone induces neuronal death; at concentrations 10–100 µmol, it potentiates neurotoxicity induced by NMDA, Fe, BSO, amyloid-β and MPP+; increased calcium influx. At concentrations of 10 µM and higher, it induces concentration-dependent inward current, significant increase in oxidative stress; NMDA iGluR antagonists attenuate Ca^2+^ influx, electrophysiological effects and toxicity, mGluR5 antagonist and free radical scavenger.	[121]
Synthetic	*Drosophila* CS *^/^***	Oral (feeding)	2, 4, 6, 8 and 10 mM	Fed for 3 days; monitored for up to 41 days.	Reduced life span, reduced climbing performance (males more susceptible), learning and memory impairment, reduction of female fertility, accumulation of BMAA in flies—preferentially in the brain; early developmental exposure has a delayed effect on neurological behavior in the adult stages.	[122]
Synthetic	Rats, Sprague–Dawley male **		10 mM	30 and 60 min	Reduced concentrations of taurine, serine and glycine, and increased concentrations of alanine and ammonium in the brain tissue.	[123]
Synthetic	Rats, Sprague–Dawley*	s.c.	400 mg/kg	108 days	Unilateral hind-limb splay in males, whole body tremors in females, in both sexes failure to identify and discriminate a learned odor, decreased locomotor activity, decreased exploration and increased anxiety, memory deficits—spatial learning, short term working and reference memory and long-term memory impairment (nest finding test, open field test, elevated plus maze test, inclined plane test, audiogenic response, radial arm test and water maze test.	[124]
Synthetic	Rats, Wistar NSC-34 primary culture of motoneurons **		300 µM	24 and 48 h	TRPML1 protein expression downregulated, lower lysosomal Ca^2+^ efflux, reduced lysosomal Ca^2+^ content, ER Ca^2+^ depletion, increase in the expression of p62/SQSTM1 and LC3-II and GRP78 and caspase 9 upregulation.	[125]
Synthetic	Rats, Sprague Dawley male *^/^***	Inhalation	Single dose 0.0016 mg, 1.8 mg and 3.5 mg/kg bw Total amount 0.918 μg, 1.836 μg, 679.6 μg and 1359 μg per animal	5 and 10 days	No clinical signs of toxicity; absence of detectable amounts of BMAA in liver, lung or brain, but a metabolite of the labeled BMAA was observed in these tissues.	[126]
Synthetic	NT-2, SK-N-MC, and SH-SY5Y **		1 µM to 10 mM ED_50_ for cell effects: 1430–1604 µM, depending on the cell line; ED_50_ for mitochondrial effects: 362 µM	5 days	Concentration-dependent effect on viability of cells and insolated mitochondria starting at 10 and 100 µM, respectively, effects partially blocked by NMDA, non-NMDA and mGluR1 antagonists with highest attenuation when all three are combined.	[127]
Synthetic	Rats, HSD; Mice, B6C3F1/N *^/^***	Oral (gavage) i.v.	Per os 1, 10, or 100 mg/kg	1, 5 and 10 days	L-BMAA was well absorbed following gavage administration and distributed to most tissues in rats and mice, with no affinity for accumulation in the brain, potential for accumulation of L-BMAA-derived species following repeated exposure, L-BMAA-equivalents were incorporated into macromolecules in the brain.	[128]
Synthetic	Mice, CD1 female *^/^***	Oral (gavage)	15.5 g/kg	11 weeks	BMAA detectable in the brain and liver, no behavioral abnormalities, contents of dopamine, noradrenaline, serotonin or 5-hydroxyindoleacetic acid not altered in the striatum, contents of aspartate and glutamate not altered in the cortex, glycine levels significantly lower in the cortex, no pathological changes in the cerebral cortex, hippocampus, striatum, substantia nigra and spinal cord.	[129]
Washed cycad flour made from *C. circinalis* seeds and “chips”	Mice, CD1 male *^/^***	Oral (feeding)	0.5 g of cycad flour per day. A tested sample of flour contained 0.003 µg/g of BMAA	25–27 days	Progressive decline of motor function (gait length, hind leg extension, tremor of the hind limbs) that continues after cessation of Cycad flour feeding; decline of cognitive functions affecting primarily reference memory (longer latencies in water-maze and relearning tests, higher number of entry errors in radial arm test); TUNEL and caspase-3 positive staining in the cortex, hippocampus, substantia nigra and spinal cord; tau-positive cells in the cortex, hippocampus and substantia nigra; significant decrease in cortical thickness in the motor and lateral entorhinal cortices, decreased number of motor neurons in the spinal cord.	[130]
Crude extract of washed cycad flour made from *C. circinalis* seeds and “chips”	Rat neocortical slices **		1:50 dilution of crude flour extract. A tested sample of flour contained 0.003 µg/g of BMAA	2 min and 1 h	Depolarisation of field potentials and increased LDH release, both blocked by NMDA receptor antagonists.	[130]
Purified from *C. micronesica* Hill gametophyte extracts	Mice, dissociated spinal cord culture **		30, 100, 300 and 1000 µM	30 min, 24 h	Dose-dependent selective MN injury at 30 µM, widespread neurodegeneration at 1000 µM, effects blocked by non-NMDA receptor antagonists, rise of intracellular Ca^2+^ concentration and generation of ROS in all neurons, more pronounced in MNs.	[131]
*C.micronesica* Hill gametophyte extracts	Mice, dissociated spinal cord culture **		17–35 µM	24 h	Selective dose-dependent MN injury, effects blocked by non-NMDA receptor antagonists.	[131]
Synthetic	Mice cell lines BV-2 and Neuro 2A **		0.001, 0.1 and 10 µM	24, 48 and 72 h	Dose-dependent cell death, but not reaching LD_50_ level, neuroblastoma cell line more sensitive than µglial cells; BMAA did not induce apoptosis nor did it affect the TNF-alpha levels, but did cause necrosis and production of ROS in neurons.	[132]
Synthetic	Human, primary human astrocytes **		0.1 to 1000 µM	1 to 24 h	Dose-dependent LDH release from astrocytes starting at 0.1 µM BMAA, decreased cell proliferation and increase in ROS generation at IC_50_ and IC_20_; increased Ca^2+^ influx into the astrocytes at IC_50_ but not at IC_20_.	[133]
Synthetic	SH-SY5Y cells **		3, 5 and 10 mM	24 and 48 h	Dose- and time-dependent decrease of cellular viability, increase in nuclear GSK3alpha and GSK3β levels; increase in aberrant forms of TDP-43.	[134]
Synthetic	Chicken retina **		0.75-3 mM	30 min	Vacuolisation and swelling of the neuronal somas in the inner nuclear layer, the inner plexiform layer and the ganglion cell layer; effects blocked completely by NMDA iGluR antagonist.	[135]
Synthetic	Biochemical investigation on commercial enzyme kits **		650 nm, 3, 50, 88, 100, 300, 500 µM	1–6 h	Significant reduction of the β-amylase, catalase and glutathione S-transferase activity, no effect on the activity of peroxidase, superoxide dismutase and native ribonuclease H; concentration-dependent interference with protein folding in absence of de novo protein synthesis.	[136]
Synthetic	Mice, dissociated mouse cortical culture **		1 and 3 mM	1 h	Acute swelling and substantial late degeneration of cortical neurons, release of LDH to the medium, rapid membrane depolarization and increased membrane conductance; all effects attenuated by NMDA iGLuR antagonists and produced only in presence of bicarbonate ions.	[137]
Synthetic	Rats, Wistar male *^/^***	i.p.	100 to 350 mg/kg/day	Administered for 5 days; analyzed after 90–100 days.	Dose-dependent neurological damage (ambulation, hind-foot reflex, strength); disassembly of endoplasmatic reticulum, vacuolisation of the mitochondria, abundance of caspase-3 in spinal motor neurons; N-acetylaspartate (NAA) increased in both spinal cord and cortex; increase in GSK3β levels in spinal cord and motor cortex; increase in high molecular weight TDP-43 in motor cortex.	[138]
Synthetic	Monkey macaques (*Macaca fascicularis*) *	Oral (gavage)	100, 125, 200, 250, 300, 315 and 350 mg/kg	Administration: 100 mg/kg per day for 12 weeks; 250 mg/kg per day for 2 weeks plus 125 mg/kg per day for 5 to 7 weeks; 200 mg/kg per day for 6 to 10 weeks, 200 mg/kg per day for 6 to 10 weeks plus 250 to 350 mg/kg per day for 7 weeks; or 300 to 315 mg/kg per day for 2.5 to 3 weeks, plus 200 mg/kg per day for 6 weeks; followed for 12–17 weeks.	Early neurological signs: wrist-drop, clumsiness, and difficulty in picking up small objects; muscle weakness and loss of muscle bulk; unilateral or bilateral extensor hind-limb posturing; late neurological signs (after 4 weeks): stooped posture, unkempt coat, tremor in and weakness of upper or all four extremities, and a reduction or loss of characteristic aggressive behavior; disinterest in the environment and changes in normal diurnal pattern of vigilance; periods of immobility with an expressionless face and blank stare, a crouched posture and a bradykinetic, shuffling, bipedal gait; animals treated with an oral L-dopa showed selective recovery of marked facial muscle movement and spontaneous activity. Chromatolisis, somatic and dendritic swelling, neurofillament aggregates in cortical Betz cells and large anterior horn cells of the spinal cord.	[139]
Synthetic	Rats, Wistar hippocampal primary and stem cell neuronal cultures **		50, 100, 250, 500 μM, 1, 2 and 3 mM	24 h and 7 days	In primary hippocampal neurons: decreased cell viability and apoptosis or apoptosis/necrosis starting at 3 mM BMAA; in stem cells: decreased cell viability and apoptosis or apoptosis/necrosis starting at 250 µM BMAA, increased AIF translocation, reduced differentiation, reduction in the neurite length, the number of processes per cell and the number of branches per cell; all these effects inheritable and present in daughter cell cultures; decreased DNA methylation.	[140]
Synthetic	Rats, Wistar culture of olfactory ensheathing cells **		100, 500 µM, 1 and 3 mM	30 min, 1 h and 48 h	Dose-dependent increase in LDH release from glial cells; increased mitochondrial activity, Ca^2+^ influx and ROS generation in glial cells.	[141]
Environmental HABs of unknown species	*Drosophila* *^/^***	Inhalation	1.7 and 7.2 ng/L in water	Exposed for 2 h; monitored for up to 100 days.	Age-dependent and gender-specific decrease in climbing performance, reduced lifespan, reduction in structural and functional integrity of synapses.	[142]
Synthetic	Rats, wild type and SOD1 G93A transgenic *^/^***	Intrathecal	5 mM	Single injection at 80 days of age; analysis after 30 days.	Spinal cord motor neurons exhibit eccentric nuclei, swelling, vacuolar changes, fragmentation of processes, cytosolic aggregates of TDP-43; astrocytes in the ventral horn of spinal cord show increase in number and size, marked increase in oxidative stress, and decrease in GLT-1 labeling.	[143]
Synthetic	Rats, Wistar male *^/^***	s.c.	600 mg/kg	Two daily injections on PND 9 and 10; analysis at 2 weeks, 3 and 6 months.	Time dependent degeneration and necrosis of neurons, calcium deposits in neurons and astrogliosis confined to CA1 region of the hippocampus; intracytoplasmic fibril bundles in CA1 neurons and astrocytes, enrichment of proteins implicated in protein aggregation, antioxidant protection and energy metabolism.	[144]
Synthetic	Mice, C57BL/6 primary neuronal culture from frontal cortex; SHSY-5Y **		250 µM, 500 µM, 1 and 3 mM	48 h	Reduced O_2_ consumption in both isolated mitochondria and primary cortical cultures, reduced glycolysis and ATP production, decreased mitochondrial potential and increased ROS production, mitochondrial fragmentation and decreased mitochondrial turnover, stimulation of inflammasome NLRP3, activation of neuronal extracellular TLR4 and intracellular TLR3, increased caspase-1 activity, elevated levels of IL-1β, increased Tau phosphorylation and Aβ peptides production.	[145]
Synthetic	Mice, CD-1 male *^/^***	Oral (feeding)	28 mg/kg bw	Fed daily and tested twice a week for 30 days.	No change in motor coordination (rotarod), motor neuron-mediated reflexes (leg extension), locomotion (stride length and open field), or muscular strength (wire hang), no changes in cognitive behavior (radial arm water maze), no signs of neuronal damage or activation of death pathways in brains and spinal cords.	[146]
Synthetic	Rats, Wistar midbrain slices **		0.1, 0.3, 1, 3 and 10 mM	2 min and 30 min	In SNpc DAergic neurons: rapid application—reversible membrane depolarization, inward current mediated by mGluR1 coupled to TRP channels and AMPA receptors, increase of firing rate and increase of intracellular Ca^2+^ concentration mediated by mGluR1; prolonged exposure—reduction of spike frequency, reduction of membrane resistance, increase of intracellular Ca^2+^ concentration, cell shrinkage, cyt-c release into the cytosol and ROS production. In SNpc GABAergic neurons: inward current mediated by AMPA receptors.	[147]
Synthetic	Rats, Wistar spinal cord primary motor neuron culture **		300 µM	48 h	Reduced cell survival; neuroprotection induced by activation of NCX, SOD1 and ApoSOD1; neuroprotection induced by SOD1 and ApoSOD1 prevented by siNCX1.	[148]
Synthetic	Mice, C57BL/6 male *	s.c.	30 mg/kg bw	Daily injections for five days; followed for 72 days.	No effects on spatial learning and memory (Barnes maze), nor on exploration/anxiety (open field test), nor on recollection-like object memory (novel location recognition and novel object recognition tests).	[149]
Synthetic	Rats, Sprague Dawley *^/^***	s.c.	400 mg/kg	Injections on PND 3–7 or 10; single injection to pregnant dams on GD 14; analyzed after 120 days.	Age and gender-dependent neuronal loss in the hippocampus (CA1 and CA3), dentate gyrus, striatum (caudate nucleus, putamen, and dopaminergic neurons of substantia nigra pars compacta), prefrontal cortex, and spinal ventral horn; presence of β-amyloid plaques in the hippocampus, striatum and prefrontal cortex, neuroinflammation, reactive µglia, intracellular hyper-phosphorylated tau positive NFTs in the hippocampus, alpha-synuclein positive Lewy bodies in remaining neurons of the substantia nigra pars compacta, cytoplasmic aggregates of TDP-43 in the hippocampus, dentate gyrus, basal ganglia and spinal ventral horn.	[126]
Synthetic	Mice, C57BL/6 primary hippocampal neuronal culture **		100 µM, 500 µM, 1 and 3 mM	3, 24 or 48 h	Dose-dependent BMAA cell death, inhibition of PP2A activity, increase in hyperphosphorylation of tau.	[150]
Synthetic	Rats, Wistar male **		1 mM	2 h	mGluR5 dependent inhibition of PP2A activity, increase in hyperphosphorylation of tau.	[150]
Synthetic	Rats, Wistar *^/^***	i.c.v	2 μL of 1.3 μM BMAA	Single injection; analyzed after 1, 24, 48, 72 or 96 h.	Inhibition of PP2A activity, increase in hyperphosphorylation of tau.	[150]

*—in vivo; **—in vitro; ***—ex vivo; ^†^—toxicological parameters have been added to the table when reported and as reported by the authors of the original papers.

## Data Availability

Not applicable.

## Supplementary Materials

The following supporting information can be downloaded at: https://www.mdpi.com/article/10.3390/microorganisms10122418/s1, Table S1: Occurrence of BMAA and its isomers in environmental samples and organisms.

## Abbreviations

Aβ	amyloid beta
AchE	acetylcholinesterase
AD	Alzheimer’s disease
AEG	N-(2-aminoethyl)glycine
AIF	apoptosis inducing factor
ALS	amyotrophic lateral sclerosis
ALS/PDC	amyotrophic lateral sclerosis/Parkinsonism dementia complex
AMPA	α-amino-3-hydroxy-5-methyl-4-isoxazolepropionic acid
AOAC	Association of Official Analytical Chemists
AQC	6-aminoquinolyl-N-hydroxysuccinimidyl carbamate
BDNF	brain derived neurotrophic factor
BLC	biological loess crust
BMAA	β-N-methylamino-L-alanine
BSO	buthionine-S-R-sulfoximine
CaM	calmodulin
CDC-42	cell division control protein 42 homolog
CD50	half curative dose
COX-2	cyclooxygenase-2
cyt-c	cytochrome-c
DA	dopamine
DAB	2,4-diaminobutyric acid
DRD4	dopamine D4 receptor
DW	dry weight
ER	endoplasmic reticulum
ERK	extracellular signal-regulated kinase
GD	gestation day
GLT-1	glutamate transporter 1
GRP78	78 kDa glucose-regulated protein
GSK-3β	glycogen synthase kinase-3 beta
iGluR	ionotropic glutamate receptor
i.c.v.	intracerebroventricular
IL-1β	interleukin 1 beta
i.p.	intraperitoneal
i.v.	intravenous
JNK	c-Jun N-terminal kinase
LC3-II	microtubule-associated protein 1 light chain 3,
LDH	lactate dehydrogenase
MAMs	mitochondria-associated ER membranes
MAOA	monoamine oxidase A
MAPT	microtubule associated protein tau
mGluR	metabotropic glutamate receptor
MMP	mitochondrial membrane permeabilization
MN	motoneuron
MPP+	1-methyl-4-phenylpyridinium
MS/MS	tandem mass spectrometry
NA	noradrenaline
NDD	neurodegenerative disease
NF-κB	nuclear factor kappa B
NFT	neurofibrillary tangle
NLRP3	NLR family pyrin domain containing 3 protein
NMDA	N-methyl-D-aspartate
PD	Parkinson’s disease
PND	postnatal day
PP2A	protein phosphatase 2
ROS	reactive oxygen species
s.c.	subcutaneous
SNpc	substantia nigra pars compacta
SQSTM1/p62	lysosomal function and sequestosome-1/ubiquitin-binding protein p62
TDP-43	TAR DNA-binding protein 43
TH	tyrosine hydroxylase
TLR	Toll-like receptor
TNF-α	tumour necrosis factor alpha
TREM2	triggering receptor expressed on myeloid cells 2
TRPML1	transient receptor potential cation channel, mucolipin subfamily, member 1
UPR	unfolded protein response
UPS	ubiquitin-proteasome system
Xc−	cystine/glutamate antiporter
