# Supplementary material for: Environmental Neurotoxin β-N-Methylamino-L-alanine (BMAA) as a Widely Occurring Putative Pathogenic Factor in Neurodegenerative Diseases
Source: Microorganisms. 2022 Dec 6;10(12):2418. doi: 10.3390/microorganisms10122418 (PMC9781992; doi:10.3390/microorganisms10122418)
Supplement: Supplementary file 1 [file microorganisms-10-02418-s001.zip › microorganisms-2013079-supplementary.pdf]

microorganisms

MDPI

Table S1. Occurrence of BMAA and its isomers in environmental samples and organisms.

Group of organism/sample	Type of sample, Genus	Origin	Additional information	Reference
Cyanobacteria	Environmental cyanobacterial samples dominated by *Microcystis*	England	Detected BMAA. Samples of blooms, scums, and mats from 11 freshwater lakes and 1 brackish waterbody used for drinking water, recreation, or both.	[13]
Cyanobacteria	Environmental cyanobacterial samples dominated by *Aphanizomenon*	England		
Cyanobacteria	Environmental cyanobacterial samples dominated by *Oscillatoria*	Scotland		
Cyanobacteria	Environmental cyanobacterial samples dominated by *Microcystis* and *Gomphosphaeria*	England		
Cyanobacteria	Environmental cyanobacterial samples dominated by *Planktothrix*	Scotland		
Cyanobacteria	Environmental cyanobacterial samples dominated by *Pseudanabaena*	Scotland		
Cyanobacteria	Environmental cyanobacterial samples dominated by *Microcystis*	Wales		
Cyanobacteria	Environmental cyanobacterial samples dominated by *Planktothrix* and *Anabaena*	Scotland		
Cyanobacteria	Environmental cyanobacterial samples dominated by *Nodularia*	England		
Cyanobacteria	Environmental cyanobacterial samples dominated by *Oscillatoria*	Scotland		
Cyanobacteria	Environmental cyanobacterial samples dominated by *Aphanizomenon*	Scotland		
Cyanobacteria	Environmental cyanobacterial samples dominated by *Anabaena*	Scotland		
Cyanobacteria	Freshwater, lake Houston dominated by *Phormidium* sp.	Texas, USA	Detected AEG	[26]

Cyanobacteria	Freshwater, Cutler Marsh dominated by *Phormidium* sp.	Utah, USA	Detected AEG	
Cyanobacteria	Freshwater, Logan Pond dominated by *Anabaenopsis* sp.	Utah, USA	Detected AEG	
Cyanobacteria	Freshwater, Cutler Canyon River dominated by *Phormidium* sp., Diatoms, Green Algae	Utah, USA	Detected AEG	
Cyanobacteria	Freshwater, Benson Marina dominated by *Oscillatoria* sp., *Phormidium* sp.	Utah, USA	Detected AEG	
Cyanobacteria	Freshwater, River culture 1 dominated by *Chroococcales*	Mie Prefecture, Japan	Detected AEG	
Cyanobacteria	Freshwater, River culture 2 dominated by *Oscillatoria* sp.	Mie Prefecture, Japan	Detected AEG	
Cyanobacteria	Freshwater, River culture 3 dominated by *Oscillatoria* sp.	Mie Prefecture, Japan	Detected AEG	
Cyanobacteria	Freshwater, Altan Tevsh Spring dominated by *Oscillatoriales*/*Chroococcales*/Green Algae/Diatoms	Gobi Desert, Mongolia	Detected AEG	
Cyanobacteria	Freshwater, Tsagaan Tokhoi Spring dominated by *Oscillatoriales*/*Chroococcales*/Green Algae/Diatoms	Gobi Desert, Mongolia	Detected AEG	
Cyanobacteria	Freshwater, Khukh Ders Spring dominated by *Oscillatoriales*/Green Algae	Gobi Desert, Mongolia	Detected AEG	
Cyanobacteria	Freshwater, Mukhar Zadgai Spring dominated by *Oscillatoriales*/*Chroococcales*/Green Algae	Gobi Desert, Mongolia	Detected AEG	
Cyanobacteria	Plankton dominated by *Microcystis* spp.	Lake Taihu, China	Detected DAB	[23]

Cyanobacteria	Plankton dominated by *Microcystis* spp.	Lake Chaohu, China	Detected DAB	
Cyanobacteria	Plankton dominated by *Microcystis* spp. and *Anabaena flos-aquae*	Lake Hongzehu, China	Detected DAB	
Cyanobacteria	Lake water with cyanobacterial blooms	Montérégie, Canada	Detected BMAA, DAB and AEG	[43]
Cyanobacteria	Lake water with cyanobacterial blooms	Estrie, Canada	Detected BMAA, DAB and AEG	
Cyanobacteria	Lake water with cyanobacterial blooms	Abitibi Témiscamingue, Canada	Detected BMAA and DAB	
Cyanobacteria	Lake water with cyanobacterial blooms	Saguenay, Canada	Detected BMAA, DAB and AEG	
Cyanobacteria	Cyanobacterial bloom dominated by *Microcystis*	Gonghu Bay, Lake Taihu, China	Detected BMAA	[34]
Cyanobacteria	Phytoplankton of springs dominated by *Anabaena* sp.	Gobi Desert, Mongolia	Detected BMAA and DAB	[24]
Cyanobacteria	Marine water, Coastal culture dominated by *Oscillatoria* sp.	Mie Prefecture, Japan	Detected AEG	[26]
Cyanobacteria	Marine water, the Inland Sea dominated by *Lyngbia* sp.	Qatar	Detected AEG	
Cyanobacteria	Marine water, Biscayne Bay dominated by *Lyngbia* sp.	Florida, USA	Detected AEG	
Cyanobacteria	Plankton samples dominated by cyanobacteria	Askö, Baltic Sea (outside the island)	Detected BMAA	[9]
Cyanobacteria	Plankton samples dominated by cyanobacteria	Baltic Sea (open water)	Detected BMAA	
Cyanobacteria	Plankton dominated by *Nodularia spumigena* and *Aphanizomenon* sp.	Baltic Sea, Sweden	Detected BMAA and DAB	[27]

Cyanobacteria	Cyanobacterial scum from urban water dominated by *Anabaena* sp.	Nijmegen, Netherlands	Detected BMAA	[25]
Cyanobacteria	Cyanobacterial scum from urban water dominated by *Planktothrix agardhii*	Bergen-op-Zoom, Netherlands	Detected BMAA	
Cyanobacteria	Cyanobacterial scum from urban water dominated by *Microcystis aeruginosa*	Schijndel, Netherlands	Detected BMAA	
Cyanobacteria	Cyanobacterial scum from urban water dominated by *Microcystis aeruginosa*	Etten Leur, Netherlands	Detected BMAA	
Cyanobacteria	Cyanobacterial scum from urban water dominated by *Anabaena*	Budel, Netherlands	Detected BMAA	
Cyanobacteria	Cyanobacterial scum from urban water dominated by *Aphanizomenon fl os-aquae*	St-Oedenrode 1, Netherlands	Detected BMAA	
Cyanobacteria	Cyanobacterial scum from urban water dominated by *Planktothrix agardhii*	St-Oedenrode 2, Netherlands	Detected BMAA	
Cyanobacteria	Cyanobacterial scum from urban water dominated by *Planktothrix agardhii*	Tilburg 1, Netherlands	Detected BMAA and DAB	
Cyanobacteria	Cyanobacterial scum from urban water dominated by *Woronichinia naegeliana*	Arnhem, Netherlands	Detected DAB	
Cyanobacteria	Cyanobacterial scum from urban water dominated by *Microcystis aeruginosa*	Beek en Donk, Netherlands	Detected BMAA	
Cyanobacteria	Cyanobacterial scum dominated by *Anabaena*	Lake, Netherlands	Detected BMAA	[28]
Cyanobacteria	Cyanobacterial scum dominated by *Planktothrix rubescens*	Lake, Netherlands	Detected BMAA	

Cyanobacteria	Cyanobacterial mats	East Antarctica	Detected BMAA in 1 sample, DAB in 6 samples, AEG in 6 samples. Analyzed 7 samples.	[39]
Cyanobacteria	Lagoon periphyton	Thau lagoon, France	Detected BMAA, DAB and AEG	[10]
Cyanobacteria	Lagoon seston	Thau lagoon, France	Detected BMAA and DAB	
Phytoplankton	Phytoplankton of the Baltic Sea	Landsort Deep, Baltic Sea (open sea)	Detected BMAA	[44]
Water	Eutroficated lake water	Lake Finjasjön, Sweden	Detected BMAA	[40]
Water	Lake water	Lake Winnipeg, Canada	Detected BMAA	[42]
Water	Surface water	Buffalo Pound Lake, Canada	Detected BMAA	[37]
Water	Surface water	Conestogo Lake, Canada	Detected BMAA	
Water	Surface water	Lac Saint-Augustin, Canada	Detected BMAA	
Water	Surface water	Petit lac Saint-François, Canada	Detected BMAA	
Water	Surface water	Woolwich Reservoir, Canada	Detected BMAA	
Water	Water from reservoirs	Holmes reservoir, Nebraska	Detected BMAA and DAB	[38]
Water	Water from reservoirs	Pawnee reservoir, Nebraska	Detected BMAA and DAB	
Water	Water from reservoirs	Rockford reservoir, Nebraska	Detected BMAA and DAB	
Water	Water from reservoirs	Kirkman's Cove reservoir, Nebraska	Detected BMAA and DAB	
Water	Water from reservoirs	Swan Creek reservoir, Nebraska	Detected BMAA and DAB	
Water	Water from reservoirs	Willow Creek reservoir, Nebraska	Detected BMAA and DAB	
Water	Water from reservoirs	Bluestem reservoir, Nebraska	Detected DAB	

Cyanobacteria	*Anabaena* sp. FACHB-1180	Lake Chaohu, China	Detected DAB in culture	[23]
Cyanobacteria	*Anabaena* PCC 7120	USA	Detected BMAA in culture. N.A habitat	[11]
Cyanobacteria	*Anabaena* sp. PCC 73105	Cambridge, UK	Detected AEG in axenic culture. Isolated from freshwater habitat	[26]
Cyanobacteria	*Anabaena variabilis* ATCC 29413	USA	Detected BMAA in culture. Isolated from freshwater habitat	[11]
Cyanobacteria	*Anabaena flos-aquae* SAG30.87	Canada	Detected DAB in culture	[14]
Cyanobacteria	*Aphanizomenon flos-aquae* CCAP 1401/7	N. A	Detected BMAA in culture	[28]
Cyanobacteria	*Aphanizomenon flos-aquae*	Baltic Sea	Detected BMAA in culture. Isolated from marine habitat	[11]
Cyanobacteria	*Aphanizomenon flos-aquae* FACHB-1249	Lake Chaohu, China	Detected DAB in culture	[23]
Cyanobacteria	*Aphanizomenon flos-aquae* FACHB-1290	Dianchi, China	Detected DAB in culture	
Cyanobacteria	*Calothrix sp.* FACHB-154	Changde, China	Detected DAB in culture	
Cyanobacteria	*Calothrix sp.* FACHB-167	Wuchang, China	Detected DAB in culture	
Cyanobacteria	*Calothrix* PCC 7103	N. A	Detected BMAA in culture. N.A habitat	[11]
Cyanobacteria	*Calothrix crustacea* CCAP1410/9	N. A	Detected DAB in cultures	[29]
Cyanobacteria	*Chlorogloeopsis* PCC 6912	India	Detected BMAA in culture. Isolated from soil habitat	[11]
Cyanobacteria	*Chroococcidiopsis* sp. PCC 6712	California, USA	Detected AEG in axenic culture. Isolated from freshwater habitat	[26]
Cyanobacteria	*Chroococcidiopsis indica* GQ2-7	N. A	Detected BMAA in culture. Isolated from marine coral habitat	[11]
Cyanobacteria	*Chroococcidiopsis indica* GT-3-26	N. A	Detected BMAA in culture. Isolated from marine rock habitat	
Cyanobacteria	*Cyanobium* sp.	River estuarie, Portugal	Detected BMAA in culture	[30]
Cyanobacteria	*Cyanobium sp.* LEGE06068	River estuarie Douro, Portugal	Detected BMAA in culture	[31]
Cyanobacteria	*Cylindrospermopsis raciborskii* ITEP18	Brazil	Detected DAB in culture	[14]

Cyanobacteria	*Cylindrospermopsis raciborskii* CR3	Australia	Detected BMAA in culture. Isolated from freshwater habitat	[11]
Cyanobacteria	*Fischerella* sp. PCC 7521	Yellowstone, USA	Detected AEG in axenic culture. Isolated from hot spring.	[26]
Cyanobacteria	*Fischerella* PCC 7521	Yellowstone, USA	Detected BMAA in culture. Isolated from Yellowstone, hot spring	[11]
Cyanobacteria	*Lyngbia* sp. PCC 8106	North Sea, Germany	Detected AEG in axenic culture. Isolated from marine habitat	[26]
Cyanobacteria	*Lyngbya majuscula*	Zanzibar	Detected BMAA in culture. Isolated from marine habitat	[11]
Cyanobacteria	*Lyngbya maiussula* Harv FACHB-866	Chengdu, China	Detected DAB in culture	[23]
Cyanobacteria	*Lyngbya cryptovaginatus* FACHB-890	China	Detected DAB in culture	
Cyanobacteria	*Leptolyngbya* PCC 73110	N. A	Detected BMAA and DAB in culture	[27]
Cyanobacteria	*Leptolyngbya* PCC 73110	N. A	Detected BMAA and AEG in culture	[32]
Cyanobacteria	*Leptolyngbya* PCC 73110	N. A	Detected DAB in culture	[29]
Cyanobacteria	*Leptolyngbya* sp.	River estuarie, Portugal	Detected BMAA in culture	[30]
Cyanobacteria	*Leptolyngbya sp. 1* LEGE06069	River estuarie Douro, Portugal	Detected BMAA in culture	[31]
Cyanobacteria	*Leptolyngbya sp. 1* LEGE07080	River estuarie Minho, Portugal	Detected BMAA in culture	
Cyanobacteria	*Leptolyngbya sp. 1* LEGE07084	River estuarie Minho, Portugal	Detected BMAA in culture	
Cyanobacteria	*Leptolyngbya sp. 1* LEGE07091	River estuaries Vouga, Portugal	Detected BMAA in culture	
Cyanobacteria	*Leptolyngbya sp. 2* LEGE06070	River estuarie Douro, Portugal	Detected BMAA in culture	
Cyanobacteria	*Leptolyngbya sp. 2* LEGE 07075	River estuarie Douro, Portugal	Detected BMAA in culture	
Cyanobacteria	*Leptolyngbya aff. bijugata* LEGE07085	River estuarie Douro, Portugal	Detected BMAA in culture	
Cyanobacteria	*Microcoleus* sp.	River estuarie, Portugal	Detected BMAA in culture	[30]

Cyanobacteria	*Microcoleus vaginatus* LEGE07076	River estuarie Minho, Portugal	Detected BMAA in culture	[31]
Cyanobacteria	*Microcoleus chthonoplastes* LEGE07092	River estuarie Vouga, Portugal	Detected BMAA in culture	
Cyanobacteria	*Microcystis* PCC 7806	The Netherlands	Detected BMAA in culture. Isolated from freshwater habitat	[11]
Cyanobacteria	*Microcystis* PCC 7820	Scotland	Detected BMAA in culture. Isolated from freshwater habitat	
Cyanobacteria	*Microcystis aeruginosa* AB2005/26	China, Lake Chaohu	Detected DAB in culture	[14]
Cyanobacteria	*Microcystis aeruginosa* AB2005/28	China, Lake Chaohu	Detected DAB in culture	
Cyanobacteria	*Microcystis aeruginosa* AB2005/30	China, Lake Chaohu	Detected DAB in culture	
Cyanobacteria	*Microcystis aeruginosa* AB2005/31	China, Lake Chaohu	Detected DAB in culture	
Cyanobacteria	*Microcystis aeruginosa* AB2005/32	China, Lake Chaohu	Detected DAB in culture	
Cyanobacteria	*Microcystis aeruginosa* AB2005/33	China, Lake Chaohu	Detected DAB in culture	
Cyanobacteria	*Microcystis aeruginosa* AB2005/40	China, Lake Chaohu	Detected DAB in culture	
Cyanobacteria	*Microcystis aeruginosa* AB2005/45	China, Lake Chaohu	Detected DAB in culture	
Cyanobacteria	*Microcystis aeruginosa* AB2005/46	China, Lake Chaohu	Detected DAB in culture	
Cyanobacteria	*Microcystis aeruginosa* Terramare	Germany	Detected DAB in culture	
Cyanobacteria	*Microcystis aeruginosa* FACHB-315	N. A	Detected DAB in culture. Strain purchased from the freshwater algae collection in the Institute of Hydrobiology (FACHB), Chinese Academy of Sciences	[23]
Cyanobacteria	*Microcystis aeruginosa* FACHB-905	N. A	Detected DAB in culture. Strain purchased from the freshwater algae collection in the Institute of Hydrobiology (FACHB), Chinese Academy of Sciences	
Cyanobacteria	*Myxosarcina* sp. LEGE 06146	Portugal	Detected BMAA in culture. Isolated from marine habitat	[35]
Cyanobacteria	*Myxosarcina burmensis* GB-9-4	Marshall Islands, USA	Detected BMAA in culture. Isolated from marine coral habitat	[11]
Cyanobacteria	*Myxosarcina concinna* GT-7-6	N. A	Detected BMAA in culture. Isolated from marine coral habitat	

Cyanobacteria	*Nostoc* sp. LEGE 06077	Portugal	Detected BMAA in culture. Isolated from estuarine habitat	[35]
Cyanobacteria	*Nostoc* sp. PCC 7120	N. A	Detected AEG in axenic culture	[26]
Cyanobacteria	*Nostoc* PCC 9305	N. A	Detected BMAA in culture. Isolated from symbiosis with *Anthoceros*	[11]
Cyanobacteria	*Nostoc* PCC 7422	N. A	Detected BMAA in culture. Isolated from symbiosis with *Cycas*	
Cyanobacteria	*Nostoc* PCC 9229	N. A	Detected BMAA in culture. Isolated from symbiosis with *Gunnera monoica*	
Cyanobacteria	*Nostoc* PCC 6310	Israel	Detected BMAA in culture. Isolated from freshwater habitat	
Cyanobacteria	*Nostoc* PCC 7107	USA	Detected BMAA in culture. Isolated from freshwater habitat	
Cyanobacteria	*Nostoc* Pc	N. A	Detected BMAA in culture. Isolated from symbiosis with *Peltigera*	
Cyanobacteria	*Nostoc* PCC 7107	N. A	Detected DAB in culture	[29]
Cyanobacteria	*Nostoc sp.* FACHB-106	Changsha, China	Detected DAB in culture	[23]
Cyanobacteria	*Nostoc* sp. FACHB-973	Lake Donghu, China	Detected DAB in culture	
Cyanobacteria	*Nostoc* 29150	N. A	Detected BMAA, AEG and DAB in culture	[36]
Cyanobacteria	*Nostoc*	Kauai, Hawaii	Detected BMAA in axenic culture. Strain isolated from roots of *C. micronesica from* National Tropical Botanical Garden (Kalaheo, Kauai, Hawaii)	[8]
Cyanobacteria	*Nostoc* enc	N. A	Detected BMAA in culture. Isolated from symbiosis with *Encephalartos*	[11]
Cyanobacteria	*Nostoc* 8001	N. A	Detected BMAA in culture. Isolated from symbiosis with *Gunnera monoica*	
Cyanobacteria	*Nostoc* 8963	N. A	Detected BMAA in culture. Isolated from symbiosis with *Gunnera prorepens*	
Cyanobacteria	*Nostoc* 8964	N. A	Detected BMAA in culture. Isolated from symbiosis with *Gunnera macrophylla*	

Cyanobacteria	*Nostoc sp.* CMMED 01	Hawaiian Islands	Detected BMAA in culture. Isolated from marine habitat	
Cyanobacteria	*Nostoc* 268	Baltic Sea	Detected BMAA in culture. Isolated from brackish Water	
Cyanobacteria	*Nostoc* CCMP 2511	N. A	Detected DAB in culture	[29]
Cyanobacteria	*Nostoc endophytum* CCAP1453/14	N. A	Detected DAB in culture	
Cyanobacteria	*Nostoc* sp.	River estuarie, Portugal	Detected BMAA in culture	[30]
Cyanobacteria	*Nostoc sp.* LEGE06077	River estuarie Minho, Portugal	Detected BMAA in culture	[31]
Cyanobacteria	*Nostoc* sp.	Guam	Detected AEG. Cultured cycad root endosymbiont	[26]
Cyanobacteria	*Nodularia* sp. PCC 73104	British Columbia, Canada	Detected AEG in axenic culture. Isolated from terrestrial environment	
Cyanobacteria	*Nodularia spumigena* Huebel 1988/306	Germany	Detected DAB in culture	[14]
Cyanobacteria	*Nodularia harveyana* Huebel 1983/300	Germany	Detected DAB in culture	
Cyanobacteria	*Nodularia spumigena* NSGG0205	Poland	Detected DAB in culture	
Cyanobacteria	*Nodularia spumigena*	Baltic Sea	Detected BMAA in culture. Isolated from brackish water	[11]
Cyanobacteria	*Nodularia harveyana* CCAP 14521	N. A	Detected BMAA in culture. Isolated from marine habitat	
Cyanobacteria	*Nodularia* sp.	River estuarie, Portugal	Detected BMAA in culture	[30]
Cyanobacteria	*Nodularia sp.* LEGE06071	River estuarie Vouga, Portugal	Detected BMAA in culture	[31]
Cyanobacteria	*Oscillatoria* sp. PCC 6506	N. A	Detected AEG in culture	[26]
Cyanobacteria	*Phormidium*	N. A	Detected BMAA in culture. N.A habitat	[11]
Cyanobacteria	*Phormidium* sp.	River estuarie, Portugal	Detected BMAA in culture	[30]
Cyanobacteria	*Phormidiumcf.animale* LEGE06072	River estuarie Vouga, Portugal	Detected BMAA in culture	[31]
Cyanobacteria	*Phormidium cf. chalybeum* LEGE06078	River estuarie Douro, Portugal	Detected BMAA in culture	
Cyanobacteria	*Planktothrix agardhii* NIES 595	Northern Ireland	Detected BMAA in culture. Isolated from freshwater habitat	[11]

Cyanobacteria	*Planktothrix agardhii* FACHB-1243	Lake Chaohu, China	Detected DAB in culture	[23]
Cyanobacteria	*Planktothrix agardhii*, FACHB-1261	Lake Taihu, China	Detected DAB in culture	
Cyanobacteria	*Plectonema PCC* 73110	N. A	Detected BMAA in culture. N.A habitat	[11]
Cyanobacteria	*Prochlorococcus marinus* CCMP1377	Sargasso Sea	Detected BMAA in culture. Isolated from marine habitat	
Cyanobacteria	*Symploca* PCC 8002	N. A	Detected DAB in culture	[29]
Cyanobacteria	*Symploca* PCC 8002	UK	Detected BMAA in culture. Isolated from marine, intertidal habitat	[11]
Cyanobacteria	*Scytonema* PCC 7110	Bermuda	Detected BMAA in culture. Isolated from limestone cave	
Cyanobacteria	*Scytonema sp.* FACHB-626	China	Detected DAB in culture	[23]
Cyanobacteria	*Synechococcus sp.* TES 206V	Thau lagoon, France	Detected DAB in culture	[10]
Cyanobacteria	*Synechococcus sp.* TES 206R	Thau lagoon, France	Detected DAB in culture	
Cyanobacteria	*Synechococcus sp.* TES 206H6	Thau lagoon, France	Detected DAB in culture	
Cyanobacteria	*Synechococcus sp.* TES 206D8	Thau lagoon, France	Detected DAB in culture	
Cyanobacteria	*Synechococcus elongatus* CCAP1479/1B	N. A	Detected DAB in culture	[29]
Cyanobacteria	*Synechococcus sp.* FACHB-1061	Changjiang River, China	Detected DAB in culture	[23]
Cyanobacteria	*Synechococcus* sp.	River estuarie, Portugal	Detected BMAA in culture	[30]
Cyanobacteria	*Synechococcus sp.* LEGE07074	River estuarie Douro, Portugal	Detected BMAA in culture	[31]
Cyanobacteria	*Synechococcus* PCC 6301	U.S. A	Detected BMAA in culture. Isolated from freshwater habitat	[11]
Cyanobacteria	*Synechocystis* sp. CC 6803	California, USA	Detected AEG in axenic culture. Isolated from freshwater habitat	[26]
Cyanobacteria	*Synechocystis* sp.	River estuarie, Portugal	Detected BMAA in culture	[30]
Cyanobacteria	*Synechocystis salina* LEGE06079	River estuarie Douro, Portugal	Detected BMAA in culture	[31]
Cyanobacteria	*Synechocystis cf. salina* LEGE06083	River estuarie Douro, Portugal	Detected BMAA in culture	
Cyanobacteria	*Synechocystis cf. salina* LEGE07073	River estuarie Vouga, Portugal	Detected BMAA in culture	

Cyanobacteria	*Trichodesmium thiebautii*	Caribbean	Detected BMAA in culture. Isolated from marine habitat	[11]
Cyanobacteria	*Trichodesmium* CCMP1985	coastal North Carolina	Detected BMAA in culture. Isolated from marine habitat	
Cyanobacteria	N.A strain	Kauai, Hawaii	Detected BMAA in culture. Strain isolated from infected coralloid roots of *Cycas micronesica*	[41]
Cyanobacteria-diatoms	planktonic field samples dominated by *Leptolyngbya* sp./*Naviculales*	Kristineberg, Swedish west coast	marine, planktonic field samples	[33]
Cyanobacteria based supplements	*Aphanizomenon flos-aquae* capsules	Lake Klamath, OR, USA	Detected DAB in one supplement	[14]
Cyanobacteria based supplements	*Aphanizomenon flos aquae* dietary supplements	N. A	Detected BMAA	[46]
Cyanobacteria based supplements	*Aphanizomenon flos aquae* dietary supplements	Klamath Lake, USA	Detected BMAA	
Cyanobacteria based supplements	*Spirulina* containing supplements (10 supplements)	N. A	Detected AEG in 9 supplements, detected DAB in ten supplements. Analyzed 10 supplements	[45]
Cyanobacteria based supplements	*Spirulina* containing natural health products	Canada	Detected BMAA in 4 supplements, detected DAB and AEG in 5 supplements	[19]
Cyanobacteria based supplements	*Spirulina* powder from commercial raw ingredient producers	N. A	Detected BMAA, DAB and AEG	[36]
Biocrusts	Cyanobacterial biocrusts	Al Dawodiyu, Qatar	Detected BMAA	[48]
Biocrusts	Cyanobacterial biocrusts	Al Dawodyu, Qatar	Detected DAB and AEG	[49]

Biocrusts	Cyanobacterial biocrusts	Al Kharrara, Qatar	Detected BMAA, DAB and AEG	
Biocrusts	Cyanobacterial biocrusts	Al Kharrara, Qatar	Detected DAB and AEG	[50]
Biocrusts	Biocrusts	Al Kharrara, Qatar	Detected BMAA, DAB and AEG	[47]
Aerosol	Air-filters from the lake shore	Lake Mascoma, USA	Detected BMAA, DAB and AEG	[51]
Aerosol	Air-filters from the lake shore	Goose Pond, USA	Detected DAB	
Diatoms	*Achnanthes* sp. CCAP 1095/1	Millport, Scotland	Detected BMAA in axenic culture. Isolated from marine habitat	[33]
Diatoms	*Aulacoseira*	Nepean River, Australia	Detected BMAA, AEG and DAB in cultures. Isolated from freshwater habitat	[55]
Diatoms	*Asterionellopsis glacialis* CCMP 139	N. A	Detected DAB and AEG in culture	[54]
Diatoms	*Chaetoceros calcitrans*	N. A	Detected BMAA and DAB in culture	[10]
Diatoms	*Chaetoceros* sp.	Argenton, France	Detected BMAA and DAB in culture	
Diatoms	*Chaetoceros pumilum*	Argenton, France	Detected DAB in culture	
Diatoms	*Chaetoceros calcitrans* CCMP 1315	N. A	Detected BMAA and DAB in non-axenic culture	[56]
Diatoms	*Chaetoceros* sp.	Argenton, France	Detected BMAA and DAB in non-axenic culture	
Diatoms	*Cyclotella*	Lake Liddell, Australia	Detected BMAA and DAB in cultures. Isolated from freshwater	[55]
Diatoms	*Fragilaria*	Murrumbidgee River, Australia	Detected AEG and DAB in cultures. Isolated from freshwater habitat	
Diatoms	*Halamphora coffeaeformis* CCAP 1001/2	N. A	Detected DAB and AEG in culture	[54]
Diatoms	*Navicula*	Lostock Dam, Australia	Detected BMAA, AEG and DAB in cultures. Isolated from freshwater habitat	[55]
Diatoms	*Navicula pelliculosa* CCAP 1050/9	Massachusetts, USA	Detected BMAA in axenic culture. Isolated from marine habitat	[33]
Diatoms	*Odontella aurita* AC 815	N. A	Detected DAB and AEG in culture	[54]
Diatoms	*Pseudo-nitzschia delicatissima*	France	Detected DAB in culture	

Diatoms	*Phaeodactylum tricornutum*	N. A	Detected BMAA and DAB in culture	[10]
Diatoms	*Phaeodactylum tricornutum* CCAP 1055/1	N. A	Detected BMAA and DAB in non-axenic and axenix cultures	[56]
Diatoms	*Proboscia inermis* CCAP 1064/1	Brandsfield Strait	Detected BMAA in axenic culture. Isolated from marine habitat	[33]
Diatoms	*Skeletonema marinoi* SAAE08603	Gullmarsfjorden, Sweden	Detected BMAA in axenic culture. Isolated from marine habitat	
Diatoms	*Skeletonema marinoi* ST28	Stromstad, Sweden	Detected BMAA in axenic culture. Isolated from marine habitat	
Diatoms	*Skeletonema marinoi*	N. A	Detected DAB in culture	[10]
Diatoms	*Skeletonema pseudocostatum*	Bouin, France	Detected DAB and AEG in culture	
Diatoms	*Thalassiosira pseudonana* CCMP 1015	N. A	Detected BMAA and DAB in non-axenic culture	[56]
Diatoms	*Thalassiosira pseudonana*	N. A	Detected BMAA and DAB in culture	[10]
Diatoms	*Thalassiosira weiss flogii*	N. A	Detected DAB in culture	
Diatoms	*Thalassiosira* CCAP 1085/15	Loch Linnhe, UK	Detected BMAA in axenic culture. Isolated from marine habitat	[33]
Diatoms	*Tabellaria*	Spencers Creek, Australia	Detected BMAA, AEG and DAB in cultures. Isolated from freshwater habitat	[55]
Dinoflagellates	*Alexandrium catenella* C2-4	Thau lagoon, France	Detected DAB and AEG in culture	[10]
Dinoflagellates	*Alexandrium catenella* C11-4	Thau lagoon, France	Detected DAB and AEG in culture	
Dinoflagellates	*Alexandrium minutum*	France	Detected DAB in culture	[54]
Dinoflagellates	*Gymnodinium catenatum*	Sesimbra, Portugal	Detected BMAA, AEG and DAB in culture	[57]
Dinoflagellates	*Heterocapsa triquetra*	France	Detected DAB in culture	[54]
Dinoflagellates	*Prorocentrum micans*	France	Detected DAB and AEG in culture	
Dinoflagellates	*Pyrocystis noctulica* CCMP 732	N. A	Detected DAB and AEG in culture	
Dinoflagellates	*Scrippsiella trochoïdea*	France	Detected DAB in culture	
Dinoflagellates	*Symbiodinium microadriaticum* CCMP 828	N. A	Detected DAB in culture	
Green algae	*Dunaliella salina* CCAP 19/18	N. A	Detected DAB in culture	

Green algae	*Chlamydomonas reginae* CCAP 11/78	N. A	Detected DAB and AEG in culture	
Green algae	*Chlorella vulgaris* CCAP 211/25	N. A	Detected DAB and AEG in culture	
Green algae	*Ostreococcus tauri*	Thau lagoon, France	Detected DAB and AEG in culture	[10]
Euglenas	*Eutreptiella gymnastica* CCMP 1594	N. A	Detected DAB in culture	
Red algae	*Porphyridium purpureum* CCAP 1380/5	N. A	Detected DAB and AEG in culture	
Haptophyta	*Emiliana huxleyi* CCMP 371	N. A	Detected DAB in culture	
Haptophyta	*Tisochrysis lutea* CCAP 927/14	N. A	Detected DAB in culture	
Cryptophyta	*Hemiselmis* sp. RCC 659	N. A	Detected DAB in culture	
Cryptophyta	*Proteomonas* sp. RCC 3072	N. A	Detected DAB in culture	
Cryptophyta	*Rhinomonas* sp. RCC 821	N. A	Detected DAB in culture	
Cryptophyta	*Rhodomonas salina* RCC 1506	N. A	Detected DAB in culture	
Cryptophyta	*Rhodomonas* sp. RCC 1978	N. A	Detected DAB in culture	
Cryptophyta	Unidentified *cryptophyceae*	France	Detected DAB in culture	
Zooplankton	Zooplankton of the Baltic Sea	Landsort Deep, Baltic Sea (open sea)	Detected BMAA	[44]
Zooplankton	Zooplankton samples of the Baltic Sea	Baltic sea (open water)	Detected BMAA	[9]
Bivalves	*Anodonta woodiana*	Gonghu Bay, Lake Taihu, China	Detected BMAA	[34]
Bivalves	*Antigona lamellaris*	Beihai City, Chine	Detected DAB	[59]
Bivalves	*Atrina pectinata*	Dalian City, China	Detected DAB	
Bivalves	*Arca inflata*	Lianyungang City, China	Detected DAB	
Bivalves	*Arca inflata*	Qinhuangdao City, China	Detected DAB	
Bivalves	*Cerastoderma edule*	Rio de Aveiro, Portugal	Detected BMAA, DAB and AEG	[57]
Bivalves	*Cerastoderma edule*	Ria Formosa, Portugal	Detected BMAA, DAB and AEG	
Bivalves	*Chlamys farreri*	Rongcheng City, China	Detected DAB	[59]
Bivalves	*Chlamys farreri*	Dalian City, China	Detected DAB	
Bivalves	*Chlamys farreri*	Lianyungang City, China	Detected DAB	
Bivalves	*Chlamys farreri*	Qingdao City, China	Detected DAB	
Bivalves	*Corbicula fluminea*	Gonghu Bay, Lake Taihu, China	Detected BMAA	[34]

Bivalves	*Crassosstrea gigas*	France	Detected BMAA in tissue, farmed sea-food	[60]
Bivalves	*Crassostrea gigas*	Thau lagoon, France	Detected BMAA, DAB and AEG	[29]
Bivalves	*Crassostrea gigas*	French coast, France	Detected BMAA and DAB in digestive gland tissues	[54]
Bivalves	*Crassostrea virginica*	Louisiana	Detected BMAA, Brackish habitat	[61]
Bivalves	*Crassostrea virginica*	Mississippi	Detected BMAA, Brackish habitat	
Bivalves	*Crassostrea* sp.	Beihai City, Chine	Detected DAB	[59]
Bivalves	*Crassostrea* sp.	Zhoushan City, China	Detected DAB	
Bivalves	*Crassostrea* sp.	Rongcheng City, China	Detected DAB	
Bivalves	*Crassostrea* sp.	Ningde City, China	Detected DAB	
Bivalves	*Crassostrea* sp.	Wenzhou City, China	Detected DAB	
Bivalves	*Crassostrea* sp.	Dalian City, China	Detected DAB	
Bivalves	*Crassostrea* sp	Lianyungang City, China	Detected DAB	
Bivalves	*Crassostrea* sp	Qinhuangdao City, China	Detected DAB	
Bivalves	*Crassostrea* sp	Qingdao City, China	Detected DAB	
Bivalves	*Gafrarium tumidum*	Shenzhen City, China	Detected DAB	
Bivalves	*Mactra chinensis*	Rongcheng City, China	Detected DAB	
Bivalves	*Mactra chinensis*	Dalian City, China	Detected DAB	
Bivalves	*Moerella iridescens*	Wenzhou City, China	Detected DAB	
Bivalves	*Mercenaria mercenaria*	Lianyungang City, China	Detected DAB	
Bivalves	*Mytilus edulis*	Sweden (west coast)	Detected BMAA in tissue, farmed sea-food	[60]
Bivalves	*Mytilus edulis*	Europe (Scandinavia)	Detected BMAA	[62]
Bivalves	*Mytilus edulis platensis*	South America	Detected BMAA	
Bivalves	*Mytilus edulis*	Kattegat Sea, Sweden (west coast)	Detected BMAA	[9]
Bivalves	*Mytilus edulis*	French coast, France	Detected BMAA and DAB in digestive gland tissues	[54]
Bivalves	*Mytilus galloprovincialis*	Thau lagoon, France	Detected BMAA, DAB and AEG	[10]
Bivalves	*Mytilus galloprovincialis*	Thau lagoon, France	Detected BMAA, DAB and AEG	[29]
Bivalves	*Mytilus galloprovincialis*	Zhoushan City, China	Detected DAB	[59]

Bivalves	*Mytilus galloprovincialis*	Rongcheng City, China	Detected DAB	
Bivalves	*Mytilus galloprovincialis*	Ningde City, China	Detected DAB	
Bivalves	*Mytilus galloprovincialis*	Dalian City	Detected DAB	
Bivalves	*Mytilus galloprovincialis*	Lianyungang City, China	Detected DAB	
Bivalves	*Mytilus galloprovincialis*	Qinhuangdao City, China	Detected DAB	
Bivalves	*Mytilus galloprovincialis*	Qingdao City, China	Detected DAB	
Bivalves	*Mytilus galloprovincialis*	French coast, France	Detected BMAA and DAB in digestive gland tissues	[54]
Bivalves	*Mytilus coruscus*	Zhoushan City, China	Detected BMAA and DAB	[59]
Bivalves	*Mytilus coruscus*	Wenzhou City, China	Detected DAB	
Bivalves	*Ostrea edulis*	Greece	Detected BMAA in tissue, farmed sea-food	[60]
Bivalves	*Ostrea edulis*	Sweden (west coast)	Detected BMAA in tissue, farmed sea-food	
Bivalves	*Ostrea edulis*	Kattegat Sea, Sweden (west coast)	Detected BMAA	[9]
Bivalves	*Ostrea edulis*	Stockholm, Sweden	Detected BMAA and DAB. Bought in a fish market in Stockholm, Sweden.	[27]
Bivalves	*Perna viridis*	Beihai City, Chine	Detected DAB	[59]
Bivalves	*Perna viridis*	Shenzhen City, China	Detected DAB	
Bivalves	*Perna canaliculus*	Australia	Detected BMAA	[62]
Bivalves	*Periglypta petechialis*	Beihai City, Chine	Detected DAB	[59]
Bivalves	*Placopecten magellanicus*	North America	Detected BMAA	[62]
Bivalves	*Ruditapes philippinarum*	Rongcheng City, China	Detected DAB	
Bivalves	*Ruditapes philippinarum*	Ningde City	Detected DAB	
Bivalves	*Ruditapes philippinarum*	Dalian City, China	Detected DAB	
Bivalves	*Ruditapes philippinarum*	Qingdao City, China	Detected DAB	
Bivalves	*Ruditapes philippinarum*	Lianyungang City, China	Detected DAB	[59]
Bivalves	*Solen strictus*	Rongcheng City, China	Detected BMAA	
Bivalves	*Sinonovacula constricta*	Ningde City, China	Detected DAB	
Bivalves	*Sinonovacula constricta*	Wenzhou City, China	Detected DAB	
Bivalves	*Sinonovacula constricta*	Lianyungang City, China	Detected DAB	

Bivalves	*Scapharca subcrenata*	Dalian City, China	Detected DAB	
Bivalves	*Tegillarca granosa*	Wenzhou City, China	Detected DAB	
Bivalves	Mussel	West Coast, Sweden	Detected BMAA	[63]
Gastropods	*Bellamya aeruginosa*	Gonghu Bay, Lake Taihu, China	Detected BMAA	[34]
Gastropods	*Volutharpa ampullacea*	Dalian City, China	Detected DAB	
Gastropods	*Haliotis discus hannai*	Ningde City, China	Detected DAB	
Gastropods	*Neverita didyma*	Rongcheng City, China	Detected BMAA and DAB	
Gastropods	*Neverita didyma*	Laizhou City, China	Detected BMAA and DAB	
Gastropods	*Neverita didyma*	Qingdao City, China	Detected BMAA and DAB	
Gastropods	*Neverita didyma*	Dalian City, China	Detected BMAA and DAB	[59]
Gastropods	*Neverita didyma*	Lianyungang City, China	Detected BMAA and DAB	
Gastropods	*Neptunea cumingii*	Dalian City, China	Detected DAB	
Gastropods	*Natica maculosa*	Ningde City, China	Detected DAB	
Gastropods	*Rapana venosa*	Qinhuangdao City, China	Detected DAB	
Gastropods	*Rapana venosa*	Qingdao City, China	Detected DAB	
Crustaceans	*Callinectes sapidus*	Florida	Detected BMAA, Marine habitat	[61]
Crustaceans	*Cancer pagurus*	Ireland, Northeast Atlantic	Detected BMAA	[62]
Crustaceans	*Callinectes sapidus*	Chesapeake Bay, USA	Detected BMAA and DAB in claws, swimming muscle, hepatopancreas and hepatopancreas under heart. DAB detected in the heart	[64]
Crustaceans	*Callinectes sapidus*	Chesapeake Bay, USA	Detected BMAA, DAB and AEG	[36]
Crustaceans	*Eriocheir sisensis*	Gonghu Bay, Lake Taihu, China	Detected BMAA	[34]
Crustaceans	*Heterocarpus ensifer*	Sweden	Detected BMAA in tissue. Bought seafood	[60]
Crustaceans	*Heterocarpus ensifer*	Northern Antlantic	Detected BMAA in tissue. Bought seafood	
Crustaceans	*Macrobrachium nipponense*	Gonghu Bay, Lake Taihu, China	Detected BMAA	[34]

Crustaceans	*Mysis mixta*	Landsort Deep, Baltic Sea (open sea)	Detected BMAA	[44]
Crustaceans	*Neomysis integer*	Landsort Deep, Baltic Sea (open sea)	Detected BMAA	
Crustaceans	*Palaemon modestus* Heller	Gonghu Bay, Lake Taihu, China	Detected BMAA	[34]
Crustaceans	*Procambarus clarkii*	Gonghu Bay, Lake Taihu, China	Detected BMAA	
Crustaceans	*Panulirus* sp.	Florida	Detected BMAA and DAB in lobsters' tails	[65]
Fish	*Abramis brama*	Lake Finjasjön, Sweden	Detected BMAA in brain and muscle	[40]
Fish	*Aristichthys nobilis*	Gonghu Bay, Lake Taihu, China	Detected BMAA	[34]
Fish	*Anguilla anguilla*	Lake Finjasjön, Sweden	Detected BMAA in brain	[40]
Fish	*Carassius auratus*	Gonghu Bay, Lake Taihu, China	Detected BMAA	[34]
Fish	*Carcharhinus acronotus*	Biscayne Bay, South Florida	Detected BMAA in shark fins	[66]
Fish	*Carcharhinus acronotus*	Atlantic Ocean	Detected BMAA in shark fins	[37]
Fish	*Carcharhinus limbatus*	Biscayne Bay, South Florida	Detected BMAA in shark fins	[66]
Fish	*Carcharhinus limbatus*	Florida Bay, South Florida	Detected BMAA in shark fins	
Fish	*Carcharhinus limbatus*	Atlantic Ocean	Detected BMAA in shark fins	[67]
Fish	*Carcharhinus leucas*	Florida Bay, South Florida	Detected BMAA in shark fins	[66]
Fish	*Carcharhinus leucas*	Atlantic Ocean	Detected BMAA in shark fins	[67]
Fish	*Coregonus lavaretus*	Stockholm, Sweden	Detected BMAA. Bought in a fish market in Stockholm, Sweden.	[27]
Fish	*Coregonus lavaretus*	Baltic sea	Detected BMAA in muscle and brain tissues	[9]
Fish	*Coilia ectenes taihuensis*	Gonghu Bay, Lake Taihu, China	Detected BMAA	[34]
Fish	*Clupea harengus*	Baltic sea	Detected BMAA in tissue. Bought seafood	[60]

Fish	*Clupea harengus*	Baltic sea	Detected BMAA in muscle and brain tissues	[9]
Fish	*Cyprinus carpio*	Gonghu Bay, Lake Taihu, China	Detected BMAA	[34]
Fish	*Cyprinus carpio*	Lake Mascoma, USA	Detected BMAA in brain, liver and muscle, detected DAB in brain	[51]
Fish	*Erythroculter ilishaeformis*	Gonghu Bay, Lake Taihu, China	Detected BMAA	[34]
Fish	*Esox lucius*	Lake Finjasjön, Sweden	Detected BMAA in brain and muscle	[40]
Fish	*Ginglymostoma cirratum*	Biscayne Bay, South Florida	Detected BMAA in shark fins	[66]
Fish	*Ginglymostoma cirratum*	Florida Bay, South Florida	Detected BMAA in shark fins	
Fish	*Ginglymostoma cirratum*	Atlantic Ocean	Detected BMAA in shark fins	[67]
Fish	*Gymnocephalus cernua*	Lake Finjasjön, Sweden	Detected BMAA in brain and muscle	[40]
Fish	*Galeocerdo cuvier*	Pacific Ocean	Detected BMAA in shark fins	[67]
Fish	*Hemiramphus kurumeus*	Gonghu Bay, Lake Taihu, China	Detected BMAA	[34]
Fish	*Hypophthalmichthys molitrix*	Gonghu Bay, Lake Taihu, China	Detected BMAA	
Fish	*Neosalanx taihuensis*	Gonghu Bay, Lake Taihu, China	Detected BMAA	
Fish	*Negaprion brevirostris*	Florida Bay, South Florida	Detected BMAA in shark fins	[66]
Fish	*Negaprion brevirostris*	Atlantic Ocean	Detected BMAA in shark fins	[67]
Fish	*Osmerus eperlanus*	Baltic sea	Detected BMAA in muscle and brain tissues	[9]
Fish	*Parabramis pekinensis*	Gonghu Bay, Lake Taihu, China	Detected BMAA	[34]
Fish	*Pelteobagrus fulvidraco*	Gonghu Bay, Lake Taihu, China	Detected BMAA	
Fish	*Parasilurus asotus*	Gonghu Bay, Lake Taihu, China	Detected BMAA	
Fish	*Perca fluviatilis*	Lake Finjasjön, Sweden	Detected BMAA in brain and muscle	[40]

Fish	*Pleuronectes platessa*	Northeast Atlantic	Detected BMAA in tissue. Bought seafood	[60]
Fish	*Pleuronectes platessa*	Baltic sea	Detected BMAA in tissue. Bought seafood	
Fish	*Protosalanx hyalocranius*	Gonghu Bay, Lake Taihu, China	Detected BMAA	[34]
Fish	*Pseudorasbora parva*	Gonghu Bay, Lake Taihu, China	Detected BMAA	
Fish	*Rutilus rutilus*	Lake Finjasjön, Sweden	Detected BMAA in brain and muscle	
Fish	*Rhodeus sinensis*	Gonghu Bay, Lake Taihu, China	Detected BMAA	
Fish	*Rhizoprionodon terraenovae*	Atlantic Ocean	Detected BMAA in shark fins	[67]
Fish	*Sander lucioperca*	Lake Finjasjön, Sweden	Detected BMAA in brain and muscle	[40]
Fish	*Salvelinus alpinus*	Sweden	Detected BMAA in tissue. Farmed seafood	[60]
Fish	*Scophthalmus maximus*	Baltic sea	Detected BMAA in muscle and brain tissues	[9]
Fish	*Sphyrna tiburo*	Florida Bay, South Florida	Detected BMAA in shark fins	[66]
Fish	*Sphyrna tiburo*	Atlantic Ocean	Detected BMAA in shark fins	[67]
Fish	*Sphyrna mokarran*	Biscayne Bay, South Florida	Detected BMAA in shark fins, kidney, liver, muscle	[66]
Fish	*Sphyrna mokarran*	Florida Bay, South Florida	Detected BMAA in shark fins, kidney, liver, muscle	
Fish	*Sphyrna mokarran*	Atlantic Ocean	Detected BMAA in shark fins	[67]
Fish	*Sphyrna zygaena*	Atlantic Ocean	Detected BMAA in shark fins	
Fish	*Tinca tinca*	Lake Finjasjön, Sweden	Detected BMAA in brain and muscle	[40]
Fish	*Triglopsis quadricornis*	Baltic sea	Detected BMAA in brain tissue	[9]
Fish	Carp	Kirkman's Cove, Nebraska	Detected BMAA and DAB	[38]
Fish	Carp	Rockford, Nebraska	Detected BMAA and DAB	
Fish	Carp	Swan Creek, Nebraska	Detected BMAA and DAB	
Fish	Carp	Willow Creek, Nebraska	Detected BMAA and DAB	
Fish	White crappie	Kirkman's Cove, Nebraska	Detected BMAA	

Fish	White crappie	Pawnee, Nebraska	Detected BMAA and DAB	
Fish	White crappie	Rockford, Nebraska	Detected BMAA and DAB	
Fish	Bass	Pawnee, Nebraska	Detected BMAA and DAB	
Fish	Bass	Rockford, Nebraska	Detected BMAA and DAB	
Fish	Bass	Willow Creek, Nebraska	Detected BMAA	
Fish	Shad	Pawnee, Nebraska	Detected BMAA and DAB	
Fish	Walleye	Pawnee, Nebraska	Detected BMAA and DAB	
Fish	Walleye	Rockford, Nebraska	Detected BMAA and DAB	
Fish	White crappie	Pawnee, Nebraska	Detected BMAA and DAB	
Fish	Catfish	Rockford, Nebraska	Detected BMAA and DAB	
Fish	Wiper	Swan Creek, Nebraska	Detected BMAA	
Fish	Bluegill	Willow Creek, Nebraska	Detected BMAA	
Fish based dietary supplements	Shark cartilage powders	N.A	Detected BMAA (in 15 products/16), DAB and AEG (in all 16). Analyzed 16 products from 7 manufacturers.	[68]
Mammals	Flying Foxes	Guam, Hawaii	Detected BMAA	[69]
Mammals	Flying Foxes	Guam, Hawaii	Detected BMAA in hair and wing membrane	[41]
Mammals	Patients died from ALS-PDC	Guam, Hawaii	Detected BMAA brain tissues	[8]
Mammals	Patients died from ALS	USA	Detected BMAA in human brain tissues	[71]
Mammals	Patients died from AD	Canada	Detected BMAA brain tissues	[8]
Mammals	Patients died from AD	USA	Detected BMAA in human brain tissues	[71]
Mammals	Patients with AD	Canada	Detected BMAA in brain tissues	[41]
Mammals	Human hair	Hartbeespoort, South Africa	Detected BMAA	[72]
Mammals	Dolphins	Florida	Detected BMAA in brains in 6 of 7 dolphins	[70]
Mammals	Dolphins	Massachusetts	Detected BMAA in brains in 7 of 7 dolphins	

Plants	*Azolla filiculoides*	Kauai, Hawaii	Detected BMAA	[8]
Plants	*Brassica oleracea*	N. A	Detected DAB	[27]
Plants	*Cycas debaoensis*	Jurassic Plants Nursery (Halfmoon Bay, BC), Canada	Detected BMAA, AEG and DAB in leaves	[45]
Plants	*Cycas micronesica*	Village Yigo, Guam	Detected BMAA in seeds	[73]
Plants	*Cycas micronesica*	Montgomery Botanical Centre, Miami, US	Detected BMAA in seeds	[28]
Plants	*Cycas micronesica*	Kauai, Hawai	Detected BMAA in roots, leaf tissue, the outer seed layer, the seed sarcotesta, and female gametophyte	[41]
Plants	*Cycas micronesica*	Kauai, Hawaii	Detected BMAA in seeds and root	[8]
Plants	*Cycas revoluta*	Stockholm, Sweden	Detected BMAA in seeds	[27]
Plants	*Cycas revoluta* (seeds)	Asia	Detected BMAA and DAB. Purchased from Fesaja-Versand (Scho¨nhagen, Germany)	[14]
Plants	*Cycas revoluta*	N. A	Detected BMAA in root, stem, and leaves. Detected DAB in root. Cycad plant was purchased from a flower market in Qingdao, China	[23]
Plants	*Gunnera kauaiensi*	Kauai, Hawaii	Detected BMAA	[8]
Plants	*Lathyrus latifolius* (seeds)	Germany	Detected DAB. Purched from Kiepenkerl (Norken, Germany)	[14]
Plants	Flour prepared from the gametophyte of cycad seeds	Guam	Detected BMAA	[41]
Plants	Aquatic plants	Holmes, Nebraska	Detected BMAA and DAB	[38]
Plants	Aquatic plants	Kirkman's Cove, Nebraska	Detected BMAA and DAB	
Plants	Aquatic plants	Pawnee, Nebraska	Detected BMAA and DAB	
Plants	Aquatic plants	Rockford, Nebraska	Detected BMAA and DAB	

Plants	Aquatic plants	Swan Creek, Nebraska	Detected BMAA and DAB
Plants	Aquatic plants	Willow Creek, Nebraska	Detected BMAA and DAB
